# A Ti-MOF Decorated With a Pt Nanoparticle Cocatalyst for Efficient Photocatalytic H_2_ Evolution: A Theoretical Study

**DOI:** 10.3389/fchem.2020.00660

**Published:** 2020-08-07

**Authors:** Yeshuang Zhong, Ruihan Wang, Xin Wang, Zhien Lin, Gang Jiang, Mingli Yang, Dingguo Xu

**Affiliations:** ^1^College of Chemistry, MOE Key Laboratory of Green Chemistry and Technology, Sichuan University, Chengdu, China; ^2^Institute of Atomic and Molecular Physics, MOE Key Laboratory of High Energy Density Physics and Technology, Sichuan University, Chengdu, China; ^3^Research Center for Material Genome Engineering, Sichuan University, Chengdu, China

**Keywords:** Pt nanoparticles, photocatalytic hydrogen production, MOF, 20%-MIL-125-(SCH_3_)_2_, cocatalysts, DFT, TDDFT

## Abstract

Pt nanoparticles (NPs) are often used as cocatalysts to enhance the photocatalytic hydrogen production catalyzed by the metal organic framework (MOF) materials. The catalytic efficiency of many Pt/MOF systems can be greatly improved when Pt NPs are used as cocatalysts. In this work, the Pt/20%-MIL-125-(SCH_3_)_2_ was chosen as the template material to understand the functional role of a Pt metal cocatalyst in the catalytic process. Experimentally, the catalytic activity of Pt/20%-MIL-125-(SCH_3_)_2_ is more than 100 times that of the system without the help of Pt NPs. Firstly, we proposed a searching algorithm, which is based on the combined Monte Carlo (MC) method and principal component analysis (PCA) algorithm, to find that the most probable adsorption site of the Pt_13_ nanocluster loaded on the (001) surface of 20%-MIL-125-(SCH_3_)_2_. Next, by using density functional theory (DFT) and time-dependent density functional theory (TDDFT) methods, we revealed that the accumulation of some positive charges on the Pt_13_ cluster and proton adsorbed on the Pt_13_ cluster, which can promote the separation of photogenerated electrons and holes, thus improving the photocatalytic efficiency. This work not only provides a method to obtain the adsorption configuration of metal clusters on various MOFs but also provides a new insight into increasing photocatalytic efficiency for H_2_ production in Pt/MOF systems.

## Introduction

Solar-to-hydrogen conversion has attracted intensive attention because of its potential application for solving energy and environmental problems (Ran et al., 2018). Thanks to the abundant water resources and solar energy on earth, solar photocatalysis can be considered as a promising or environment friendly way to produce H_2_ from water. Ever since the application of titanium dioxide as a catalyst for hydrolysis (Fujishima and Honda, 1972), many studies based on titanium dioxide have been carried out (Asahi et al., 2001; Singh and Dutta, 2018; Kumaravel et al., 2019). TiO_2_ has many advantages in this field, e.g., low price, low toxicity, high stability, and so on (Kwon et al., 2008; He et al., 2018). However, owing to its large band gap, it just absorbs UV rays (λ ≤ 400 nm) that account for 4% of the total solar energy, which is insufficient for practical solar H_2_ production. In fact, in the field of sunlight-driven hydrogen production, how to achieve the efficient harvesting and conversion of the large portion of visible light (400 nm ≤ λ ≤ 800 nm) and infrared rays (λ ≥ 800 nm) always stays at the core position (Wang et al., 2019). In order to improve the utilization of sunlight, the metal organic framework (MOF) materials become the natural choice of candidates due to their adjustable ligands, metals, and multiple reaction sites. In particular, MOF can serve as a unique host matrix, which allows implementation of the desired properties by integration with different functional entities such as noble metal nanoparticles (NPs) (Xiao and Jiang, 2019). Consequently, materials based on MOF in photocatalysis field have developed greatly in recent years (Han et al., 2018; Wu et al., 2018; Xiao and Jiang, 2019).

Basically, when the photon energy is higher than or equal to the band gap of MOFs, the MOFs could generate photogenerated electrons and holes. The generated electrons will then be excited to the conduction bands, while the holes will remain in the valence bands. The electrons or holes continue to migrate to the surface of MOF for completing the photocatalytic reduction or oxidation reaction. Therefore, promoting the electron–hole separation is an important way to enhance the photocatalytic activity of MOFs. In some MOF photocatalysts, the photogenerated electrons and holes are easily recombined, which leads to a limited catalytic activity (Fang et al., 2018). A possible way to promote electron–hole separation is to form a semiconductor–metal junction (Wang et al., 2014), in which some metals could function as H_2_-evolution cocatalysts (Wang et al., 2019), e.g., Pt, Rh, Ru, and Ni. The frequently used cocatalysts are noble-metal NPs, e.g., Pt NPs (Yang et al., 2013). Many MOFs loaded with Pt NPs exhibit excellent photocatalytic hydrogen production performance (Toyao et al., 2013; He et al., 2014; Shen et al., 2015; Xiao et al., 2016; Han et al., 2018). We can find some examples in peer-reviewed literatures, e.g., Pt/Ti-MOF-NH_2_ (Toyao et al., 2013), Pt@UiO-66-NH_2_ (Xiao et al., 2016), Pt/UiO-66-NH_2_ (Xiao et al., 2016), Pt@MIL-125 (Shen et al., 2015), Al-TCPP-0.1Pt (Fang et al., 2018), Pt@UiO-66(Zr) (He et al., 2014), and Pt/20%-MIL-125-(SCH_3_)_2_ (Han et al., 2018). Despite tremendous advances achieved in this field, the applications based on the Pt/MOFs photocatalysis are still in the early stage of development. It is still a great challenge to develop more stable Pt/MOF photocatalysts with higher activity and cheaper MOFs. Understanding the mechanism of Pt/MOFs photocatalytic hydrogen production is clearly important for future photocatalyst design or refinement (Xiao and Jiang, 2019). However, rare theoretical studies were devoted to addressing the photocatalytic mechanism bearing in Pt/MOFs systems. In particular, the intrinsic relationships among the geometrical structure, electronic structure, the process of photoexcitation, and the electron transfer of Pt/MOFs systems are still not well-understood. In this work, we will try to explore the relationships between the structure and the photocatalysis performance of Pt/MOFs systems using the first-principles methods. Herein, the Pt/20%-MIL-125-(SCH_3_)_2_ is chosen as the template material (Han et al., 2018).

The 20%-MIL-125-(SCH_3_)_2_ was derived from the MIL-125 (Dan-Hardi et al., 2009), which is composed of cyclic octamers of TiO_2_ octahedra and terephthalic acid linker. By using the methylthio group (-SCH_3_) group as a functional group on the terephthalic acid linker [denoted as H_2_BDC-(SCH_3_)_2_], the methylthio functionalized 20%-MIL-125-(SCH_3_)_2_ can then be synthesized. The structures of MIL-125, 20%-MIL-125-(SCH_3_)_2_, H_2_BDC, and H_2_BDC-(SCH_3_)_2_ are all displayed in Figure S1. The Pt/20%-MIL-125-(SCH_3_)_2_ exhibited a promising H_2_ production rate as high as 3814.0 μmol g^−1^ h^−1^, which is the most visible-light photoactive MOFs material for H_2_ evolution from water so far (Han et al., 2018). On the other hand, this material shows some interesting aspects. The samples of 20%-MIL-125-(SCH_3_)_2_ and Pt/H_2_BDC-(SCH_3_)_2_ show a negligible activity for H_2_ production. On the contrary, with the presence of a Pt NP cocatalyst, the catalytic activity of Pt/20%-MIL-125-(SCH_3_)_2_ is 100 times higher than that of 20%-MIL-125-(SCH_3_)_2_. By comparing the photocatalytic hydrogen production of Pt/H_2_BDC-(SCH_3_)_2_ and Pt/20%-MIL-125-(SCH_3_)_2_, it clearly indicates that the framework of 20%-MIL-125-(SCH_3_)_2_ is essential to improve photocatalytic activity (Han et al., 2018). The Pt NPs also play an important role in the whole photocatalytic process, which can be known by comparing the photocatalytic hydrogen production of 20%-MIL-125-(SCH_3_)_2_ and Pt/20%-MIL-125-(SCH_3_)_2_. Experimentally, it is proposed that the Pt cocatalyst spatially separates electrons from the excited BDC-(SCH_3_)_2_ and the hole reacted with the electron donor TEOA (Han et al., 2018). This function could lead to the restraining of the recombination and the promoting hydrogen evolution on Pt NPs. However, it is not clear that how the Pt cocatalyst separates the electrons from the excited BDC-(SCH_3_)_2_. Moreover, by comparing the amount of H_2_ production among Pt/H_2_BDC-(SCH_3_)_2_, 20%-MIL-125-(SCH_3_)_2_, and Pt/20%-MIL-125-(SCH_3_)_2_, it might be natural to speculate that the interfacial interactions between Pt NPs and 20%-MIL-125-(SCH_3_)_2_ play a critical role in enhancing the photocatalytic efficiency.

In this work, we will pursue the role of metal cocatalysts in the whole catalytic process based on first-principles calculations. Of course, before further theoretical simulations, a reliable initial structure of the Pt cluster interacting with 20%-MIL-125-(SCH_3_)_2_ should be a prerequisite condition. However, due to the complexity of MOF surface structure, it is difficult to predict the adsorption sites of metal NPs. To tackle this problem, we then propose a strategy to obtain the most possible adsorption configuration efficiently, which is based on the Monte Carlo (MC) method and principal component analysis (PCA) algorithm. Subsequently, based on this structure, the structure optimization, electronic structure properties, charge density difference, and Bader charge analyses are carried out. Meanwhile, the absolute band edge position and photoexcitation process of 20%-MIL-125-(SCH_3_)_2_ are also studied. We believe that our simulations could provide some insights into how the interfacial characteristics affect the photocatalytic efficiency at the atomic level.

## Computational Details

### Construction of Pt Nanoparticle

Sakurai et al. (1999) have indicated that some transition-metal clusters (Fe, Ti, Zr, Nb, and Ta) with the “magic numbers” *n*, i.e., *n* = 7, 13, and 15 atoms in a given cluster, might have a higher geometric symmetry and/or electronic stability than clusters with other sizes (Lim and Wilcox, 2011). Experimentally, the Pt NPs with an average size of around 1 nm were observed on the surface of 20%-MIL-125-(SCH_3_)_2_ (Han et al., 2018). In order to construct the Pt nanoparticle with a size of about 1 nm, *n* = 13 was selected, which is further applied to investigate the interactions of a Pt nanoparticle with the surface of 20%-MIL-125-(SCH_3_)_2_. Herein, the global minimum state for Pt_13_ nanoparticle is determined with the ABCluster program (Zhang and Dolg, 2015), which is based on the artificial bee colony algorithm. Gupta potential, which is specifically designed for modeling metals using a many-body potential form, was employed to express the interactions between atoms of the nanocluster (Ma et al., 2017). The global minimum state for Pt_13_ is shown in Figure S2 with a symmetry of icosahedron (*I*_*h*_).

### Optimization for 20%-MIL-125-(SCH_3_)_2_, Pt_13_, and MOF Surface

To reduce the computational cost, the primitive cell of the 20%-MIL-125-(SCH_3_)_2_ framework was adopted in the periodic DFT calculations by using the Vienna *ab initio* Simulation Package (VASP) (Furthmüller and Kresse, 1996). For the geometry optimizations, the generalized gradient approximation (GGA) with the Perdew–Burke–Ernzerhof (PBE) functional was applied (Perdew et al., 1996). During the relaxation, the thresholds for the forces on atoms were set <0.05 eV/Å. The core–valence electron interactions were described by using the projector augmented wave (PAW) method (Kresse and Joubert, 1999). The kinetic energy cutoff for the plane-wave basis set expansion was set at 450 eV. A G-centered grid of *k*-points was used for integrations in the reciprocal space. The *k*-point mesh was 1 × 1 × 1. The electronic convergence criterion is 10^−5^ eV. To confirm the selection of *k*-point mesh, the lattice parameters for MIL-125 optimized by PBE are *a* = 18.937 Å, *b* = 18.941 Å, and *c* = 18.017 Å, which are quite close to experimental values of *a* = 18.654 Å, *b* = 18.654 Å, and *c* = 18.144 Å (Dan-Hardi et al., 2009). Furthermore, a larger *k*-point mesh of 2 × 2 × 2 was also tested in the single point calculation for the PBE-optimized MIL-125; the energy difference was found to be <10^−3^ eV/atom, which ensures the viability of the choice of *k*-point. In addition, the Pt_13_ cluster was optimized in a 14 × 15 × 16 Å box. Without loss of generality, the (001) surface of the MOF is selected in our simulations. A vacuum layer of 28 Å was added to prevent the mirror interaction of the cell. All Ti atoms on the surface were saturated with the terephthalic acid (Bonakala et al., 2018). All atoms are optimized except those atoms at the bottom layer (seen in Figure S3). To avoid possible spurious interactions caused by periodic boundary conditions, the dipole correction was further applied (Neugebauer and Scheffler, 1992; Rusu and Brocks, 2006). Other parameters are the same as the optimized 20%-MIL-125-(SCH_3_)_2_ unit cell.

The HSE06 (Krukau et al., 2006) exchange–correlation functional was used to obtain electronic properties including the electronic structure and freedom of the vacuum electron energy level (Wu et al., 2018). A 1 × 1 × 1 *k*-point mesh was applied in the HSE06 calculations (Nasalevich et al., 2016). The vacuum level was obtained by using a procedure developed by Butler et al. (2014).

### Model for the Pt_13_/20%-MIL-125-(SCH_3_)_2_ (001) Composite

Since no structure was reported for the Pt_13_/20%-MIL-125-(SCH_3_)_2_ composite, we first constructed the composite. In this work, we proposed a method to obtain the most possible adsorption state of the Pt_13_ cluster on the (001) surface based on combined MC and PCA method (Wold et al., 1987). In particular, the MC simulation was employed to obtain sufficient initial configurations, and the subsequent PCA simulation was used to extract the structure with the highest probability of occurrence from the saved configuration ensemble. The MC simulation was carried out using the RASPA-2.0 software (Dubbeldam et al., 2015). The Universal Force Field (UFF) parameters were employed for the energetic calculations (Rappé et al., 1992). The electrostatic interactions between the Pt_13_ cluster and the 20%-MIL-125-(SCH_3_)_2_ (001) surface were simulated using the point charges. The atom charges of the slab were calculated by the MEPO-QEq method (Kadantsev et al., 2013). The structures of the PBE optimized Pt_13_ and 20%-MIL-125-(SCH_3_)_2_ are adopted here. During the MC simulation, the MOF and Pt_13_ cluster are then treated as a rigid body. The new configurations are accepted or rejected following the Metropolis criteria. After one million MC steps, a total of 5,500 adsorption configurations were obtained. On the other hand, PCA is an effective multivariate mathematical technique for large-scale conformational analyses, which can shed some light into the conformation evolution upon the substrate binding. Details of the method can be found elsewhere (Balsera et al., 1996; Hess, 2000; Maisuradze et al., 2009). Based on our MC calculated configuration ensemble, the covariance matrices could be constructed. In addition, the entire ensemble must be aligned with the structure of the specified frame before the PCA was applied. The PC1 and PC2 are the first two eigenvectors of trajectory. Although PCA requires much longer simulation to achieve convergence, current calculations can partially ensure that the trajectory for PCA should be sufficiently long. To identify the preferred conformation for the Pt_13_ adsorption on the (001) surface, further free energy landscapes (FELs), μ(*PC*1, *PC*2) = −*k*_*B*_*T* ln *P*(*PC*1, *PC*2), were constructed based on two eigenvectors of PC1 and PC2. The local minima on the FEL could tell us the representative conformers during the MC running, and one configuration with the highest probability of occurrence can be located. In addition, further convergence tests were carried with longer MC iteration steps. Corresponding selected composite structures have no significant changes. Figure S4 gives the overlap presentation for all three structures with different MC steps.

## Results and Discussion

### Electronic Structure for 20%-MIL-125-(SCH_3_)_2_

The band gap calculated by PBE functional is 1.17 eV, which shows a relatively large difference from the experimental value of 2.69 eV. The PBE-calculated band gap value is much smaller than the experimental value. This phenomenon is not unique. We can observe similar results in other MOFs, e.g., UiO-66 (Hendrickx et al., 2015). Indeed, it is widely accepted that although the GGA functionals can predict the geometries and energetics of different materials very well, they usually underestimate the bandgaps of semiconductors, and thus cause some big errors (Cohen et al., 2008). We then choose HSE06 hybrid functional in the calculations of the electronic structures for 20%-MIL-125-(SCH_3_)_2_, since it usually predicts more accurate results for the bandgaps of MOFs than other GGA-based functionals (Finazzi et al., 2008; Hendon et al., 2013; Scanlon et al., 2013; Ni et al., 2018).

The calculated valence band maximum (VBM) of 20%-MIL-125-(SCH_3_)_2_ is −2.86 eV and the conduction band minimum (CBM) is −0.74 eV based on HSE06 functional, separately. Therefore, the band gap of 20%-MIL-125-(SCH_3_)_2_ is derived to be 2.12 eV. About 0.57 eV smaller than the experimental value (Han et al., 2018) can be observed. The computational error in the band energies calculations might be due to one-particle approximation employed in the HSE06 functional (Fu et al., 2018). However, it is still much better than the PBE-calculated result. The vacuum energy level of the 20%-MIL-125-(SCH_3_)_2_ can be further calculated to be 2.83 eV according to the general approach presented in Butler et al. (2014).

The next question is whether the 20%-MIL-125-(SCH_3_)_2_ can catalyze the reaction of H_2_ production from water without the presence of Pt NPs. In principle, if the CBM is located at a higher potential than the reductive potential energy of H^+^ to H_2_, the material is capable of H^+^ reduction. Meanwhile, if the VBM is lower than the oxidative potential energy of H_2_O to O_2_, the material is capable of H_2_O oxidation. A semiconductor spanning the redox potential of H_2_O could be considered as a suitable candidate for one-step excitation water splitting (Wang et al., 2019). Therefore, we need to identify the vacuum-aligned absolute positions of the VBM and CBM for the 20%-MIL-125-(SCH_3_)_2_ material with respect to the obtained vacuum energy level. From the above analyses, the vacuum-aligned absolute positions of the VBM and CBM are recalculated to be −5.69 and −3.57 eV, respectively. We further plot the vacuum aligned energy levels in Figure S5 with respect to the redox potential for water splitting reaction at pH = 7 and room temperature (298.15 K) (Wu et al., 2018). From Figure S5, it is quite clear that the CBM of 20%-MIL-125-(SCH_3_)_2_ (−3.57 eV) remains higher than the hydrogen evolution reaction (HER) level and the VBM (−5.69 eV) is lower than the oxygen evolution reaction (OER) level. Such electronic properties match the requirements as a potential photocatalyst for both HER and OER. Our simulations thus provide solid evidences that the 20%-MIL-125-(SCH_3_)_2_ can produce hydrogen from water when exposed to the solar radiation. Our simulations are then consistent with the experimental suggestions that the MOF itself can have some catalytic activities, although not too high (Han et al., 2018). On the other hand, it has been suggested that the photo-generated electrons can reduce H ^+^ into H_2_, and OH^−^ is oxidized into O_2_ with the help of excited holes (Wu et al., 2016). Of course, only calculations for the MOF might not fully explain why Pt NPs significantly enhance the catalytic performance. Further calculations including Pt NPs in the system are highly necessary.

### Model, Geometry, and Electronic Structures for Composite Systems

**Figure 1:**
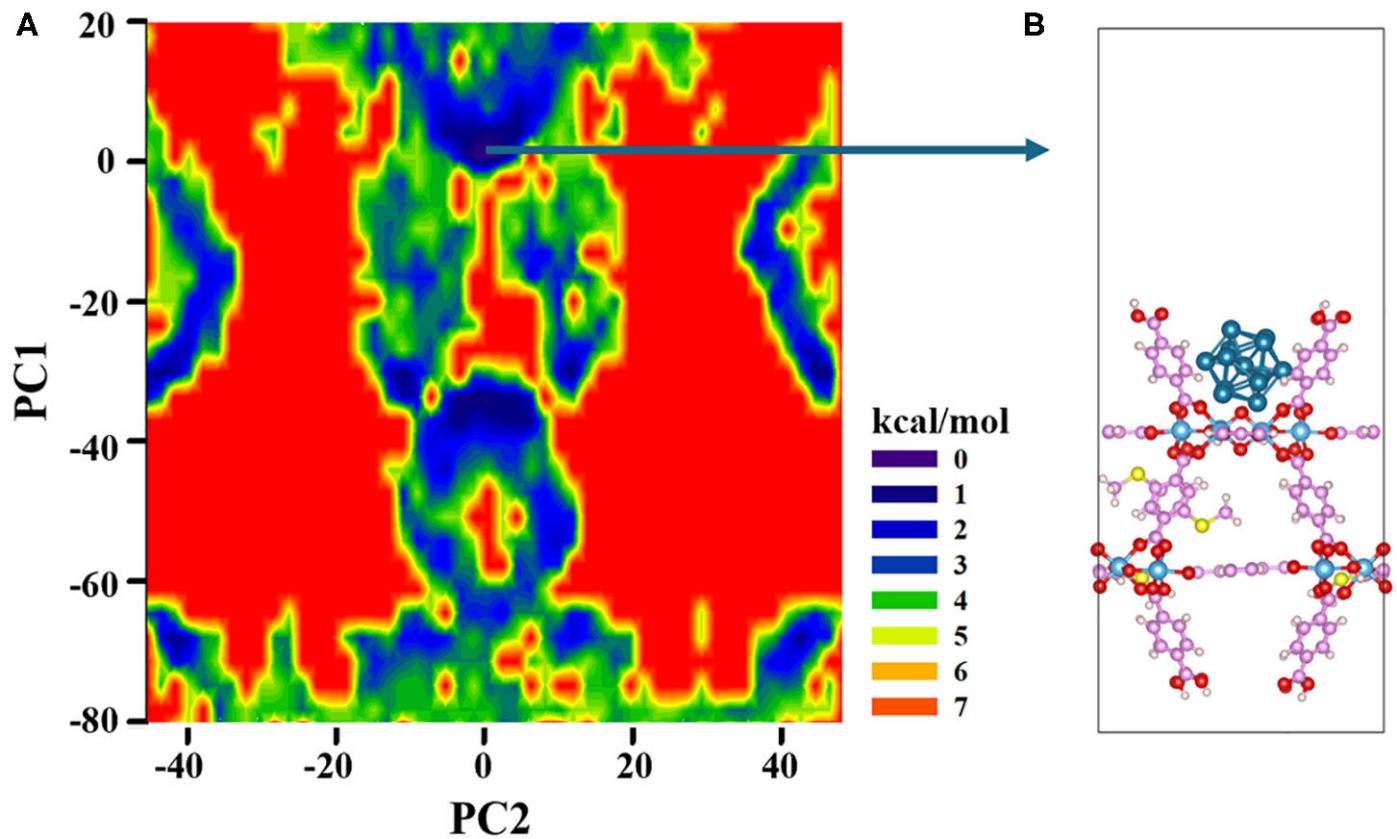
**(A)** Free energy landscapes based on PCA method. **(B)** Snapshot extracted from the MC trajectory for the Pt_13_/20%-MIL-125-(SCH_3_)_2_ (001) composite model.

It is very important to obtain a reasonable initial structure of the metal cluster interacting with MOF for subsequent calculations like catalytic mechanism. However, due to the complexity of MOF surface, it is difficult to predict the stable geometry of metal clusters interacting with MOF (Vilhelmsen et al., 2012). Herein, we develop a method to construct an initial structure of the metal clusters interacting with MOF. Of course, to build such kind of composite structures using the MOFs as the base materials, several trials have been done in peer-reviewed literatures. For example, Vilhelmsen et al. (2012) proposed a way combining the DFT and the genetic algorithm (GA) to predict the stable structure of metal clusters in MOF-74. Chen et al. (2017) applied the *ab initio* molecular dynamics (AIMD) together with annealing simulation to identify the thermodynamic stable structure of the Pd nanocluster in the UiO-66-NH_2_ pore. However, both methods are essentially computationally intensive and thus call for a cheaper method. In this work, we proposed an alternative way to obtain the composite structure more efficiently, which is based on a combined MC and PAC method. Both Pt_13_ and 20%-MIL-125(SCH_3_)_2_ (001) surface are first optimized using the PBE functional. The Pt_13_ cluster still maintains its *I*_*h*_ symmetry after optimization. The geometry of the 20%-MIL-125-(SCH_3_)_2_ (001) surface is also basically unchanged. Four terephthalic acids on the surface form a bowl-like shape, which can just accommodate a nanoparticle with the size of the Pt_13_ cluster. The next step is to identify the most probable binding structure of Pt_13_ on the MOF structure. Subsequently, to reduce the computational cost, during MC simulation, all atoms of the Pt_13_ cluster and the MOF base are then fixed at their optimized position. The PCA method is applied to reduce the three-dimensional coordinates of Pt_13_ to the two-dimensional subspace, which could reflect the position of the Pt_13_ cluster in the three-dimensional phase space. According to the free energy of Δ*G* = –*RTlnP*, a two-dimensional FEL map can be obtained, which is displayed in Figure 1A. From the landscape map, there are several dark blue areas, which relate to the states of the Pt_13_ cluster being loaded to different positions of the MOF. Such characteristics clearly indicate that one cannot build the composite structure of the Pt_13_ nanoparticle and the 20%-MIL-125-(SCH_3_)_2_ in an artificial way. These Pt_13_ cluster-loaded sites deserve further analyses. As a porous material of the 20%-MIL-125-(SCH_3_)_2_, there are basically two kinds of sites to accommodate the metal cluster of Pt_13_. Indeed, according to the size of MOF aperture vs. the size of Pt_13_, the metal cluster has the possibilities to be loaded both inside the pores and on the surface. In this work, we simply chose the structure related to the lowest energy on the FEL as the initial composite structure for Pt_13_/20%-MIL-125-(SCH_3_)_2_. According to Figure 1A, the minimum with PC1 = 0.698 and PC2 = 0.513 is selected out, which is shown in Figure 1B. The Pt_13_ cluster with an *I*_*h*_ symmetry stays right on the surface of the MOF. The shortest distance between the Pt_13_ cluster and the surface of 20%-MIL-125-(SCH_3_)_2_ is 2.84 Å. When viewed from the top, the metal cluster is located at the center of the surface as shown in Figure S6. Obviously, our model, which is the structure with the lowest total energy, is not inconsistent with experimental proposal that the Pt_13_ cluster could stay at the molecular surface of the 20%-MIL-125-(SCH_3_)_2_ (Han et al., 2018). Meanwhile, we also find that the metal cluster has the possibility to enter the pores of the supported system. Although no direct experimental data confirm this, we cannot completely exclude this. Some previous experimental studies have suggested that the metal clusters prefer to be loaded inside the pores of the porous materials with even better photocatalytic ability (Xiao et al., 2016). Nevertheless, the structure of the cluster on the MOF surface will be employed in our work for subsequent photocatalytic reactions. Overall, combining MC and PCA methods, we can quickly locate the most possible adsorption site from millions of structures. Considering the energy calculations based on the force field, we believe that our method might be an efficient way to obtain the initial composite structures, especially for those porous materials. To elucidate the whole process for the screening, a diagram is displayed in Scheme S1.

**Figure 2:**
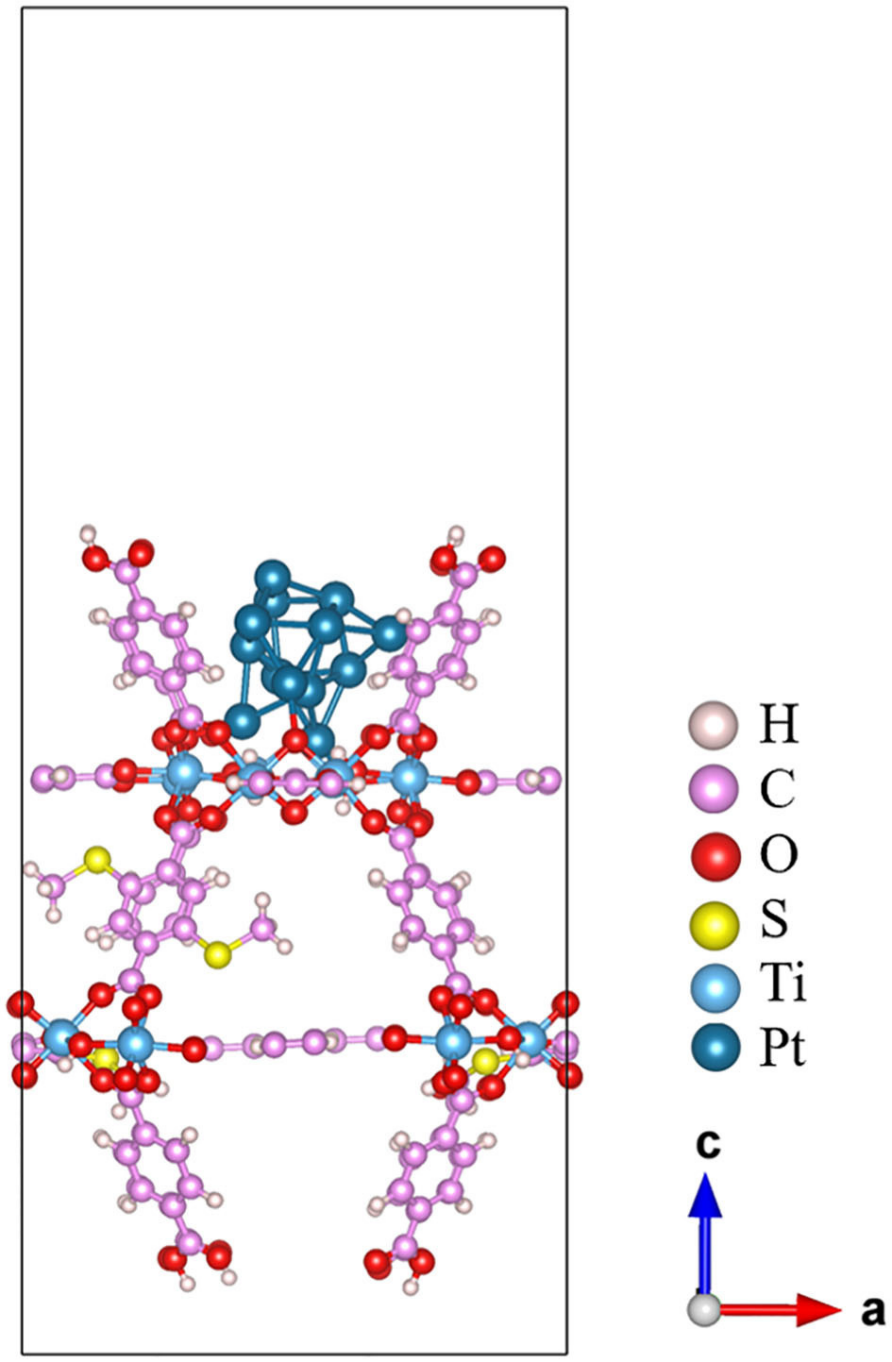
The PBE optimized structure of Pt_13_/20%-MIL-125-(SCH_3_)_2_ (001) plane.

Once the composite structure of Pt_13_/20%-MIL-125-(SCH_3_)_2_ is constructed, it is further optimized at the PBE level of theory. The corresponding optimized structure is given in Figure 2. The optimized Pt_13_ cluster features a large distortion with a low symmetry. It has a vertical height of 5.6, ~0.8 Å longer than that of the initial icosahedron Pt_13_ cluster. In addition, for the icosahedral Pt_13_, the coordination number (CN) of the central Pt atom of the cluster is 12, and the CN for the rest of the Pt atoms is 6. The Pt atoms in the optimized Pt_13_ cluster attached on the MOF surface show much lower coordination status due to structural distortion. We can see cases of CN = 3, 4, 5, and 6. The metal bond length of the Pt–Pt ranges from 2.49 to 2.76 Å, while the Pt–Pt bond length in icosahedral Pt_13_ ranges from 2.62 to 2.75 Å. On the contrary, the structure of the 20%-MIL-125-(SCH_3_)_2_ (001) surface in the composite structure can keep their original geometry with only minor distortions on a few O and H atoms, which are in direct contact with the Pt_13_ cluster. At the Pt_13_/20%-MIL-125-(SCH_3_)_2_ interfacial area, three Pt atoms at the bottom layer interact with the 20%-MIL-125-(SCH_3_)_2_ (001) surface via three Pt–O bonds. The adsorption energy of the Pt_13_ cluster on the 20%-MIL-125-(SCH_3_)_2_ (001) surface is defined as *E*_*ad*_ = *E*(*total*)−*E*(*surface*)−*E*(*Pt cluster*), which is calculated to be −4.81 eV. It suggests a relatively strong interaction between Pt_13_ cluster and the 20%-MIL-125-(SCH_3_)_2_ (001) surface.

**Figure 3:**
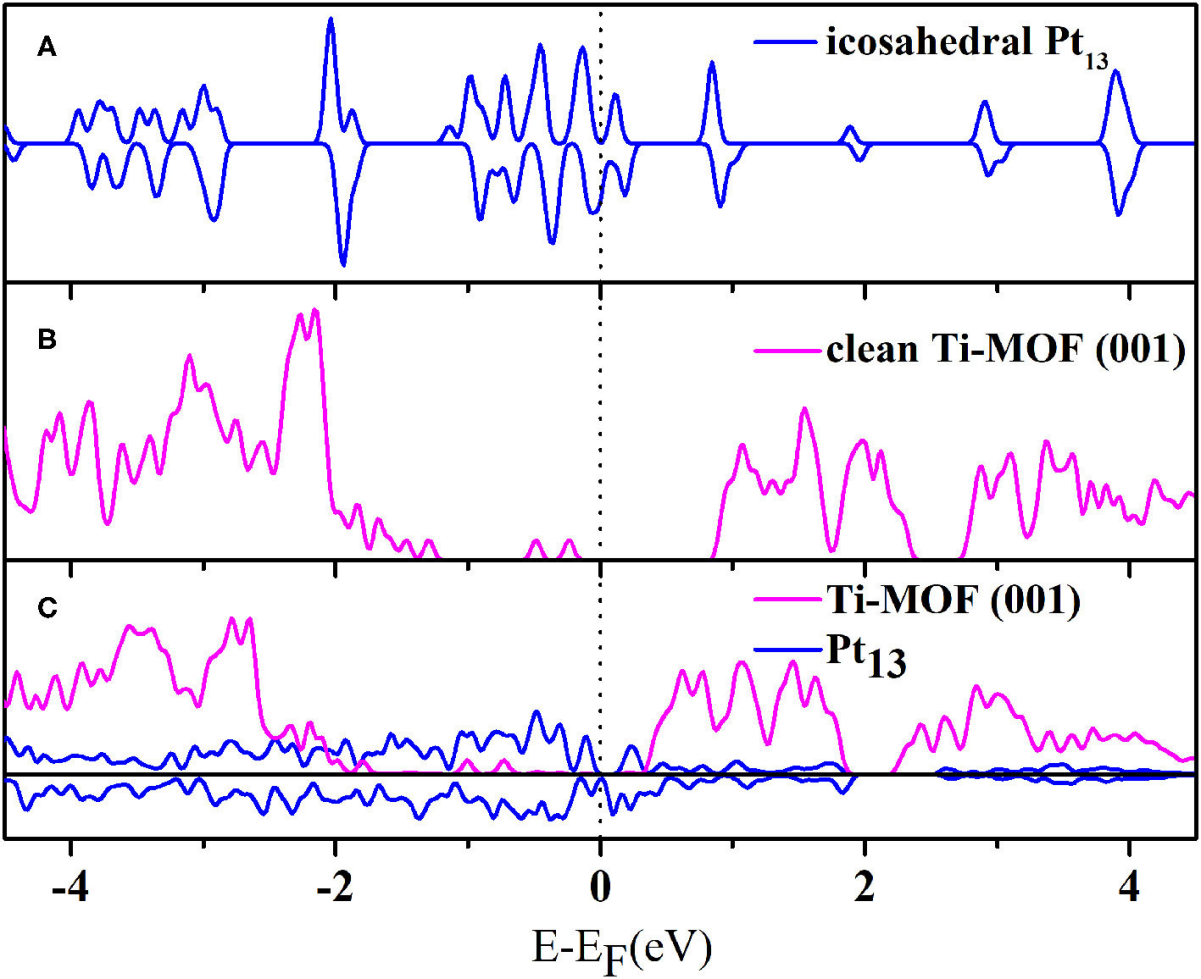
The calculated total density of states (TDOS) for **(A)** the icosahedron Pt_13_ cluster, **(B)** the clean 20%-MIL-125-(SCH_3_)_2_ (001) surface, and **(C)** the optimized Pt_13_/20%-MIL-125-(SCH_3_)_2_ system (the Pt_13_ cluster is spin-polarized).

We then analyzed the electronic structures of the systems before and after the deposition of the Pt_13_ cluster. It has been well-tested that the hybrid HSE06 functional could lead to wrong predictions for the metallic systems (Aprà and Fortunellib, 2000; Fu et al., 2015; Gao et al., 2016). This conclusion can be reproduced in our computation. In fact, the DOS calculated by HSE06 functional gives a band gap value of 0.86 eV, which indicates that the Pt_13_ cluster has semiconductor characteristics rather than those of a metal (see Figure S7 for details). This is clearly against common sense. Thus, the HSE06 functional is not appropriate to describe the electronic structure properties for the Pt_13_ cluster or the composite system. On the other hand, we have also calculated the density of states (DOS) with the PBE functional for both the Pt_13_ cluster and the optimized Pt_13_/20%-MIL-125-(SCH_3_)_2_ system. According to Figure 3A, the DOS for the Pt_13_ cluster, which is spin-polarized, gives a band gap value of 0.0 eV. The intrinsic metal characteristics of the Pt_13_ cluster can be perfectly obtained. Indeed, the spin-up DOS shows a different shape from the spin-down DOS. Hence, the spin polarization should be taken into account for the Pt_13_ cluster. Our results agree well with results presented in some peer-reviewed literatures (Yang et al., 2011; Ramos-Sanchez and Balbuena, 2013). Therefore, the PBE functional might be more appropriate for the composite system. Figures 3B,C give the calculated DOS for the 20%-MIL-125-(SCH_3_)_2_ (001) surface and the optimized Pt_13_/20%-MIL-125-(SCH_3_)_2_ system using the PBE functional. There are several interesting patterns that can be derived based on Figure 3. The DOS of the absorbed metal cluster is more continuous than that of the icosahedron Pt_13_. In addition, according to Figures 3B,C, we can also find that the loading of the Pt_13_ cluster does not induce significant changes to the shape of the DOS of 20%-MIL-125-(SCH_3_)_2_ (001) surface. The energy levels of VBM and CBM clearly have great changes, in which the VBM is far away from Fermi level, while VBM is close to Fermi level. Therefore, the loaded Pt_13_ cluster might have some effects on the MIL-125-(SCH_3_)_2_ (001) surface and cause some significant changes to the metal nanoparticle itself. We can also observe that the band gap of 20%-MIL-125-(SCH_3_)_2_ (001) surface is filled by some continuous spin-polarized Pt_13_ states. In particular, the occupied O-2p and Ti-4d states extend into the gap in the supported system to form the so-called band tail states (Wang Y. et al., 2017), which is displayed in Figure S8. The existence of the band tail states confirms again the strong interaction between the Pt_13_ cluster and the material surface. Meanwhile, they can even prevent the rapid recombination of photogenerated electrons and holes and thus facilitate the subsequent photocatalytic reaction (Chen et al., 2011).

### Electron Transfer in the Photocatalytic Reaction

Basically, a complete MOF-based photocatalytic hydrogen production process consists of three steps: (1) MOF harnesses sunlight, (2) photogenerated electrons and holes separation, and (3) reduction of protons (Xiao and Jiang, 2019). In order to improve the catalytic efficiency, some strategies have been reported. For example, Fu et al. proposed to regulate the ligands in MOF to improve light harvesting (Fu et al., 2012). The loading of cocatalysts to the MOFs is another efficient way, which can usually promote the separation of photogenerated electrons and holes (Toyao et al., 2013; He et al., 2014; Shen et al., 2015; Xiao et al., 2016). Meanwhile, the coupling hydrogen production with valuable organic oxidation was also applied to promote proton reduction (Simon et al., 2014; Kasap et al., 2016; Liu et al., 2018). For the particular system investigated in this work, Pt_13_/20%-MIL-125-(SCH_3_)_2_ composite system, the strategy based on the loading of the Pt NPs as the cocatalyst has been applied (Han et al., 2018). Experimentally, a negligible activity for H_2_ production can be observed for both 20%-MIL-125-(SCH_3_)_2_ and Pt/H_2_BDC-(SCH_3_)_2_. However, in the presence of the Pt NP cocatalyst, the catalytic activity of Pt/20%-MIL-125-(SCH_3_)_2_ is 100 times higher than that of 20%-MIL-125-(SCH_3_)_2_ (Han et al., 2018). In other words, the MIL-125-(SCH_3_)_2_ itself has poor activity to catalyze the photogenerated hydrogen production. Obviously, the involvement of Pt provides main contributions in the following reaction. Therefore, the complete understanding of the properties of the Pt/20%-MIL-125-(SCH_3_)_2_ composite structure stays at the core position, especially the electron transferring within the composite system. In this work, the electron transfer properties at the interface for the Pt_13_/20%-MIL-125-(SCH_3_)_2_ complex will then be investigated, and the process of light absorption will also be studied using the TDDFT method (Runge and Gross, 1984).

#### Electron Transfer Properties Across the Pt13/20%-MIL-125-(SCH3)2 Interface

Several methods have been proposed to characterize the electron transfer around the interfacial area, e.g., work function (Liu et al., 2016), Bader charge analysis (Lim and Wilcox, 2011; Ewing et al., 2015), and charge density difference (Wang Y. et al., 2017; Ren et al., 2018). Basically, the work function can be defined as ϕ = *E*_*vac*_−*E*_*F*_, where *E*_*F*_ is the Fermi energy and *E*_*vac*_ denotes the electrostatic potential of the vacuum level. For the system of the Pt_13_/Ti-20%-MIL-125-(SCH_3_)_2_ investigated in this work, we must obtain the work functions for the Pt_13_ cluster and Ti-20%-MIL-125-(SCH_3_)_2_ separately. However, this method might not be suitable to determine the electron transfer direction at the interface, since it cannot completely include all changes at the interface (Tung, 2000), which includes two aspects in current system. The first one is that the Pt_13_ cluster deforms greatly after contacting with the 20%-MIL-125-(SCH_3_)_2_ (001) surface. The second one is that the change in potential energy is due to the chemical interaction between the Pt_13_ cluster and the 20%-MIL-125-(SCH_3_)_2_ (001) surface (Zheng et al., 2015). To this end, we then chose Bader charge analysis and charge density difference to address the electron transfer occurring at the Pt_13_/Ti-20%-MIL-125-(SCH_3_)_2_ interface.

**Figure 4:**
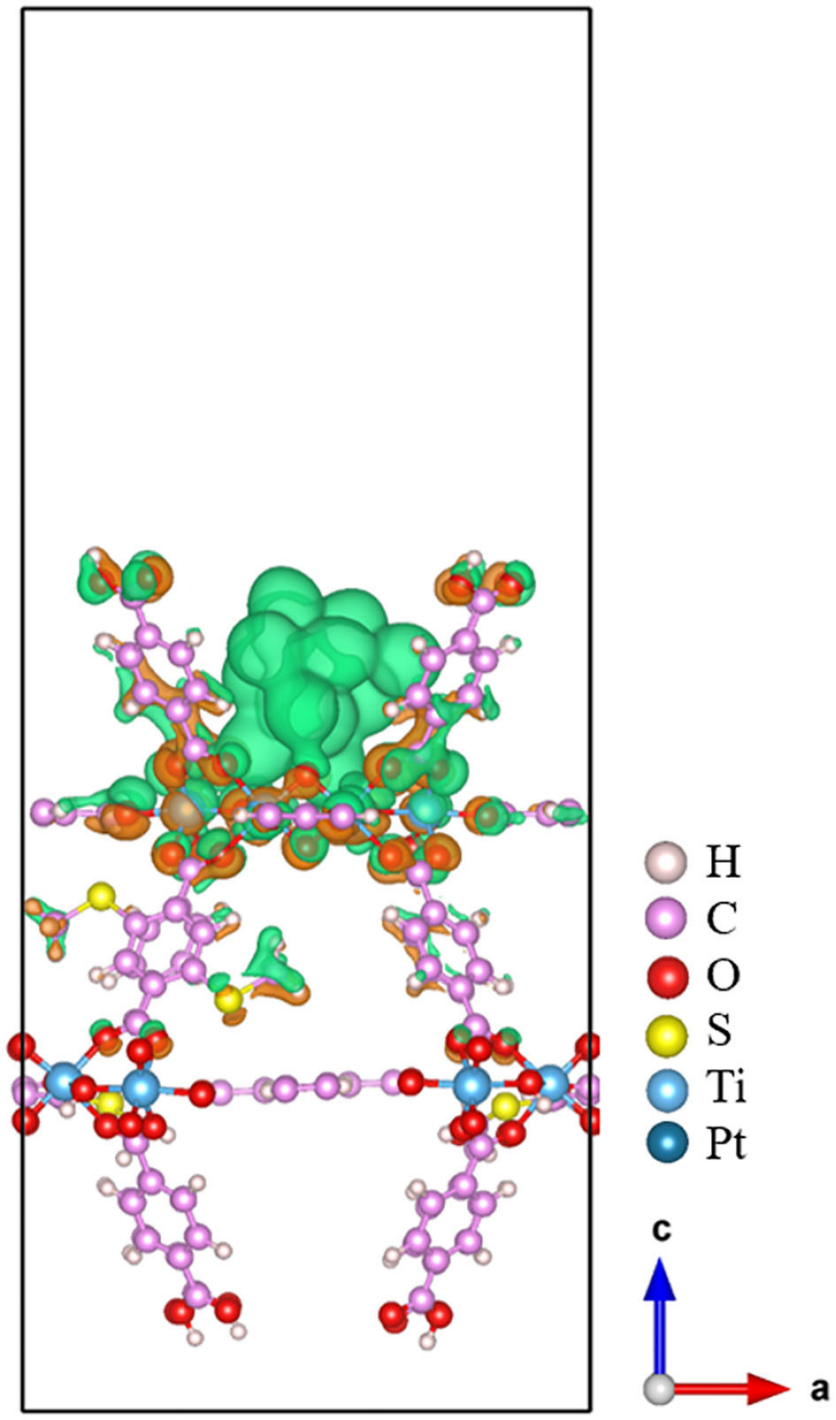
The charge density difference for Pt_13_/20%-MIL-125-(SCH_3_)_2_. The electron accumulation region is colored using the orange, while green for the electron depletion region.

**Figure 5:**
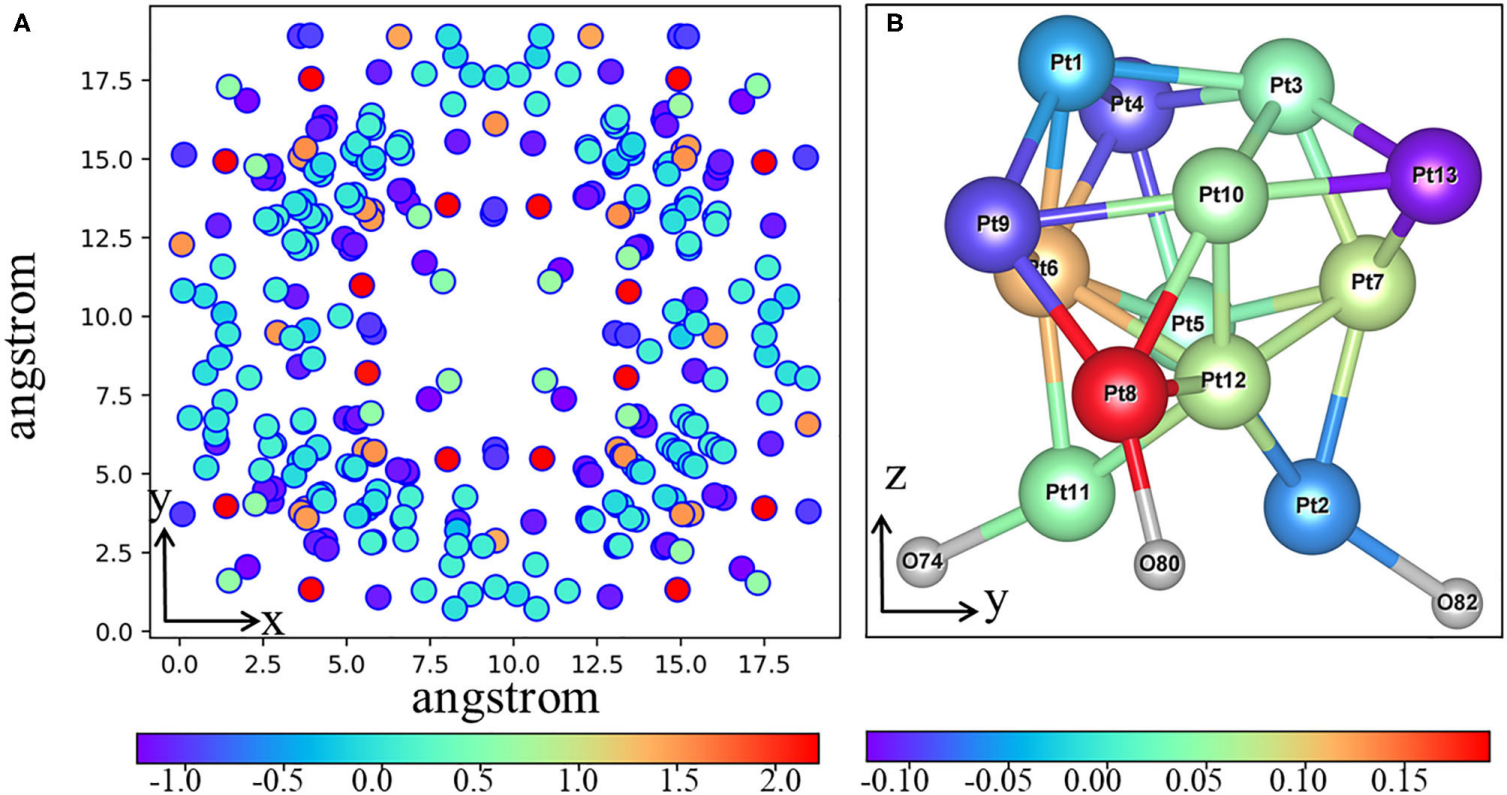
**(A)** The net Bader charges (in units of e) of the supported 20%-MIL-125-(SCH_3_)_2_ (001) surface and **(B)** adsorbed Pt_13_ in the Pt13/20%-MIL-125-(SCH_3_)_2_ (001) system. The red denotes losing an electron, and purple means getting an electron.

The three-dimensional charge density difference is depicted in Figure 4. The electron accumulation region is colored using orange, while green is used for the electron depletion region. From Figure 4, we can see that the electrons are mainly transferred from the Pt_13_ cluster to the 20%-MIL-125-(SCH_3_)_2_ (001) surface via the newly formed Pt-O bonds upon the adsorption. In addition, the Bader charge analyses can be used to obtain the amount of charge transferred across the interface quantitatively. The net charges of all individual Pt atoms are plotted in Figure 5. The corresponding calculated Bader charges and net charges of individual Pt atoms are summarized in Table S1. Total net charge on the Pt_13_ cluster is +0.20 e, which further indicates that electrons are transferred from the Pt_13_ cluster to the 20%-MIL-125-(SCH_3_)_2_ (001) surface. Clearly, consistency can be reached for two methods. Of course, such a kind of electron transfer status is not unique in the metal-support interaction systems, e.g., the composite Ru_10_/TiO_2_ system (Zhang et al., 2014). Indeed, this electron transfer was further proven to be the main factor that promotes the catalytic activity of the supported Ru_10_ cluster toward CO oxidation via reduction of the activation barrier of O and CO association. In addition, in some other systems like the Pt_13_/Graphene (Fampiou and Ramasubramaniam, 2012) and Pt_13_/Silica (Ewing et al., 2015) system, the electron migration occurs from the Pt_13_ cluster to graphene and silica, respectively. This electron transfer can also significantly impact adsorbate binding and catalytic activity (Fampiou and Ramasubramaniam, 2012; Ewing et al., 2015).

#### Light Harvesting and Electron Transfer in the Bulk Region of 20%-MIL-125-(SCH_3_)_2_

**Figure 6:**
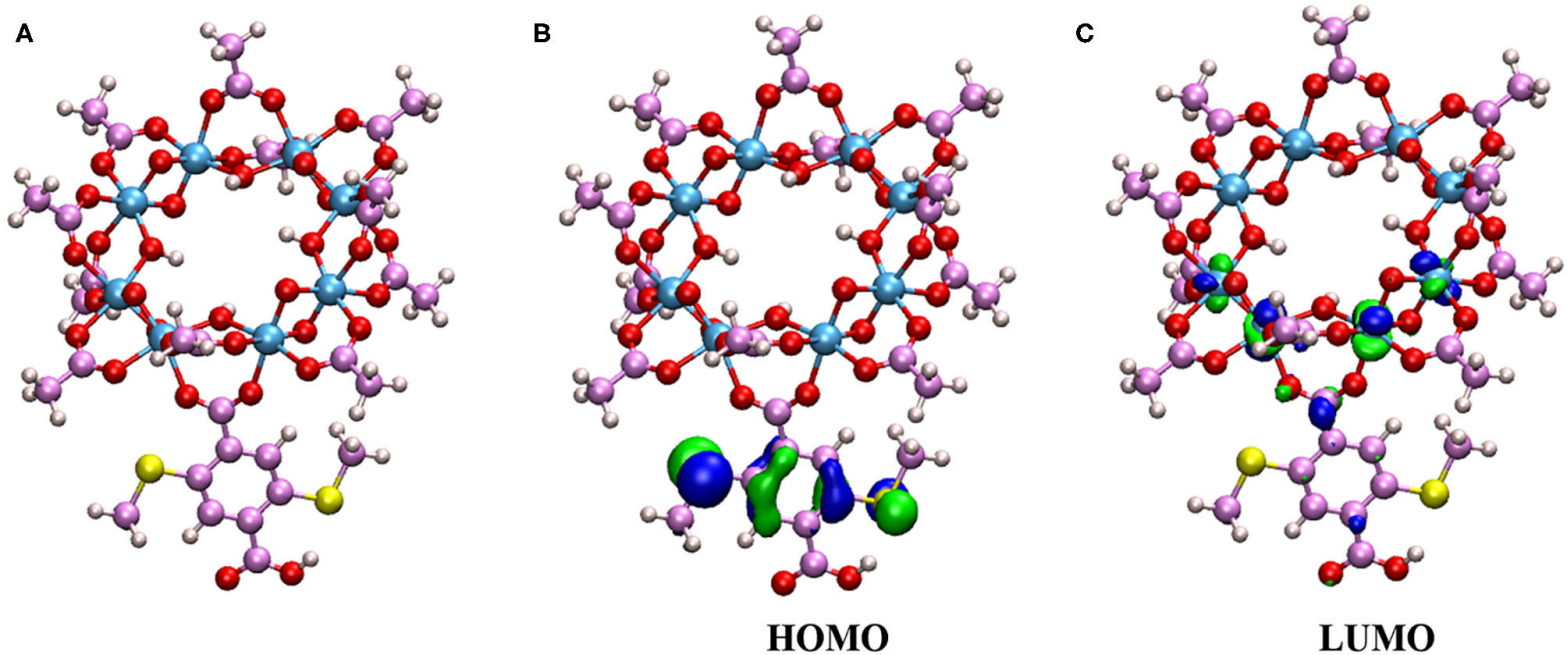
**(A)** The cluster model from the crystal for the TDDFT study, **(B)** the highest occupied molecular orbital (HOMO) of the cluster model, and **(C)** the lowest unoccupied molecular orbital (LUMO) of the cluster model.

To completely understand the electron transfer in the bulk system, we then study the generation and transfer of photogenerated electrons within the bulk of Ti-20%-MIL-125-(SCH_3_)_2_. The cluster model based on the crystal is given in Figure 6A. The model consists of 1 HBDC-(SCH_3_)_2_ unit, 1 Ti_8_O_8_(OH)_4_ unit, and 11 CH_3_COO units to avoid the terminal effects. As we have described above, the HSE06 functional was employed with the def2-TZVP basis set for C, H, O, and S atoms (Weigend and Ahlrichs, 2005), while the SDD basis set and effective core potential are used for Ti atoms (Dolg et al., 1987). The TDDFT calculations were conducted using the Gaussian 09 suite of program (Runge and Gross, 1984; Frisch et al., 2013).

A total of 10 excited states are obtained in our calculations. The first excited state has a strong oscillator strength of 0.04. This indicates that the first excited state is involved in the electronic state transition. The vertical excitation wavelength for the first excited state is 465 nm, which is close to the experimental value of 420 nm (Han et al., 2018). The small difference might be due to the discrepancy between the calculation model and the actual crystal environment. In addition, the highest occupied molecular orbital (HOMO) and the lowest unoccupied molecular orbital (LUMO) are found to play important roles in the first excitation. The molecular orbital isosurface graphs, which are obtained by Multiwfn (Lu and Chen, 2012) software, are shown in Figures 6B,C. It can be easily found that the electron density of HOMO is distributed mainly on the HBDC-(SCH_3_)_2_ unit, while the LUMO orbital is mainly distributed on the Ti atoms. Therefore, when the 20%-MIL-125-(SCH_3_)_2_ is irradiated at 420 nm, the charge will be transferred from the ligand of the HBDC-(SCH_3_)_2_ unit to Ti atoms. The Ti^4+^ will be reduced to Ti^3+^ accordingly. This process can be further assigned to a typical ligand-to-metal charge transfer (LMCT) without question.

#### Electron Transfer Promoted by Proton Adsorption on the Pt_13_ Cluster

In some metal NPs/semiconductor systems, e.g., Pt_13_/TiO_2_ system, the adsorption of proton on the metal cluster could promote electron transferring between Pt_13_ cluster and TiO_2_ (Wang D. et al., 2017). It is worthwhile to pursue how proton adsorption affects the electron transfer in the Pt_13_/20%-MIL-125-(SCH_3_)_2_ system. According to Bader charge analyses, the whole Pt_13_ cluster becomes more positively charged by the total charge changing from +0.20 e to +0.66 e along with the proton adsorption. Meanwhile, the total amount of net charge on the 20%-MIL-125-(SCH_3_)_2_ (001) slab also changes from a value of −0.2 e to a value of ~+0.30 e after the proton adsorption. Such a change suggests that the 20%-MIL-125-(SCH_3_)_2_ support attracts fewer electrons from the Pt_13_ cluster owing to the proton absorption. The adsorbed proton attracts electrons from the Pt_13_/20%-MIL-125-(SCH_3_)_2_ system, reducing itself to a net charge of −0.96 e. To avoid the artificial effects in the proton adsorption site, we then test an alternative adsorption site on the Pt_13_ cluster for comparison. Nearly the same results can be obtained in this case, but only the net charge of the Pt_13_ cluster changes to +0.76 e, while it is +0.29 e for the support material. We can safely conclude here that the proton adsorption does promote the electron transfer in the Pt_13_/20%-MIL-125-(SCH_3_)_2_ system. Corresponding adsorption site information of protons on the Pt_13_ cluster can be found in Figure S9.

#### Functional Role of the Pt_13_ Cluster in the Process of Separating the Photogenerated Electrons and Holes

Now, we can return to the critical question, i.e., why the introduction of Pt clusters can greatly improve the photocatalytic efficiency of 20%-MIL-125-(SCH_3_)_2_. From the above discussion, we can know that the Pt_13_ bears a net positive charge of +0.20 e in the Pt_13_/20%-MIL-125-(SCH_3_)_2_ system. Meanwhile, we have also revealed that the electron can be transferred from the ligand of HBDC-(SCH_3_)_2_ unit to Ti atoms upon illumination. In order to facilitate the subsequent redox reactions, it would be better for those photogenerated electrons to migrate to surface rather than the recombination of electrons and holes. In fact, for many semiconductor photocatalysts, it is quite normal that various metal ions are doped into the semiconductors to promote the separation of electrons and holes. The metal ions usually include Cu^+^ (Zhu et al., 2020), Si^4+^ (Cesar et al., 2006), Zn^2+^ (Ingler et al., 2004), and Pt^2+^ (Xing et al., 2013; Lian et al., 2017; Liu et al., 2019). For example, for the Pt-Ti^3+^/TiO_2_ system (Lian, Wang D. et al., 2017), the introduction of Pt ions greatly enhances the photocatalytic efficiency. The formation of Pt^n+^-O (*n* = 0, 2, or 3) bonds can act as the bridges to help the photogenerated electron transfer from the bulk to the surface area. Obviously, the situation is relatively similar in our system. Although the Pt atoms are not directly doped into the 20%-MIL-125-(SCH_3_)_2_, the Pt_13_ cluster could have the same function in the current system. A partial positive charge on Pt_13_ after contacting with the surface of 20%-MIL-125-(SCH_3_)_2_ can support this well. At the same time, we can also see from the Bader charge diagram (Figure 5) that there are three Pt atoms that interacted with the surface, two of which have partial positive charges. Based on the above discussion, we then speculate that the Pt_13_ cluster with a net positive charge might have similar effects with Pt atoms in the system of Pt^n+^-TiO_2_ (*n* = 0, 2, or 3). In other words, due to the accumulation of some positive charges on Pt_13_ clusters, the photogenerated electrons tend to be transferred from the bulk of 20%-MIL-125-(SCH_3_)_2_ to the surface. This would further block the recombination of photogenerated electrons and holes. Of course, the protons adsorbed on the Pt_13_ clusters might have a chance to attract electrons from the Pt_13_/20%-MIL-125-(SCH_3_)_2_ system, which further help hydrogen evolution reaction. Finally, we can conclude that the introduction of the Pt_13_ cluster to 20%-MIL-125-(SCH_3_)_2_ could dramatically change the intrinsic electronic characteristics, which leads to the improvement of photocatalytic efficiency. In order to elucidate the functional role of the Pt_13_ cluster, the electron transfer between the Pt_13_ cluster and the surface is shown in Figure S10.

## Conclusion

In recent years, the strategy of adding the metal NP cocatalyst to some MOF materials has attracted a lot of attention, since it could significantly promote the photocatalytic reactions. However, due to the complexity of the MOF surface structure, it is not easy to get the exact sites of metal NPs adsorption via both experimental and theoretical methods. We then propose an algorithm, which is based on the MC and PCA algorithm, to efficiently obtain a reliable adsorption model of metal NPs on the MOF surface. A recently reported Pt/MOF catalyst, Pt/20%-MIL-125-(SCH_3_)_2_, is chosen as the template material system in this work, since the catalytic activity of Pt/20%-MIL-125-(SCH_3_)_2_ is 100 times higher than that of 20%-MIL-125-(SCH_3_)_2_. The absolute band edge positions of VBM and CBM can confirm that the 20%-MIL-125-(SCH_3_)_2_ material has some photocatalytic activity. Subsequently, the obtained absorption model, Pt_13_/20%-MIL-125-(SCH_3_)_2_, is optimized by the first-principles methods. Bader charge and charge density difference are obtained accordingly. Results indicate that when the Pt_13_ cluster contacts the MOF surface, the electrons will transfer from the cluster to the surface of MOF, leading to some partial positive charges on the metal cluster, which is very helpful for the separation of photogenerated electrons and holes. Our research could give a hint that the introduction of some positively charged metal ions to the surface of MOF might improve the catalytic efficiency. At the same time, our calculations show that the protons absorbed on the Pt_13_ cluster can promote electron transfer from the Pt_13_/20%-MIL-125-(SCH_3_)_2_ composite to protons, so that the whole photocatalytic process can be finished. We believe that our investigations could provide some useful information for further refinement or design of the catalysts with high photocatalytic activity. Of course, some other complicated mechanistic issues are not considered here, e.g., final hydrogen evolution and the size effect of the metal clusters. More researches are on the way.

## Data Availability Statement

All datasets presented in this study are included in the article/Supplementary Material.

## Author Contributions

YZ, RW, and DX designed the study, performed the statistical analysis, and wrote the manuscript. YZ performed the first-principles calculations. RW carried out the screening of adsorption sites. DX provided research idea. XW, ZL, GJ, and MY participated in experimental guidance. All the authors read and approved the final version of the manuscript.

## Conflict of Interest

The authors declare that the research was conducted in the absence of any commercial or financial relationships that could be construed as a potential conflict of interest.

## References

[B1] Aprà E. Fortunellib A. (2000). Density-functional study of Pt_13_ and Pt_55_ cuboctahedral clusters. J. Mol. Struct. THEOCHEM 501–502, 251–259. 10.1016/S0166-1280(99)00436-4

[B2] Asahi R. Morikawa T. Ohwaki T. Aoki K. Taga Y. (2001). Visible-light photocatalysis in nitrogen-doped titanium oxides. Science 293, 269–271. 10.1126/science.1061051 11452117

[B3] Balsera M. A. Wriggers W. Oono Y. Schulten K. (1996). Principal component analysis and long time protein dynamics. J. Phys. Chem. 100, 2567–2572. 10.1021/jp9536920

[B4] Bonakala S. Lalitha A. Shin J. E. Moghadam F. Semino R. Park H. B.. (2018). Understanding of the graphene oxide/metal-organic framework interface at the atomistic scale. ACS Appl. Mater. Interfaces 10, 33619–33629. 10.1021/acsami.8b09851 30182712

[B5] Butler K. T. Hendon C. H. Walsh A. (2014). Electronic chemical potentials of porous metal-organic frameworks. J. Am. Chem. Soc. 136, 2703–2706. 10.1021/ja4110073 24447027 PMC3946036

[B6] Cesar I. Kay A. Gonzalez Martinez J. A. Gratzel M. (2006). Translucent thin film Fe_2_O_3_ photoanodes for efficient water splitting by sunlight: nanostructure-directing effect of Si-doping. J. Am. Chem. Soc. 128, 4582–4583. 10.1021/ja060292p 16594689

[B7] Chen D. L. Wu S. N. Yang P. Y. He S. H. Dou L. Wang F. F. (2017). Ab initio molecular dynamic simulations on pd clusters confined in UiO-66-NH_2_. J. Phys. Chem. C 121, 8857–8863. 10.1021/acs.jpcc.7b00957

[B8] Chen X. B. Liu L. Yu P. Y. Mao S. S. (2011). Increasing solar absorption for photocatalysis with black hydrogenated titanium dioxide nanocrystals. Science 331, 746–750. 10.1126/science.1200448 21252313

[B9] Cohen A. J. Mori-Sánchez P. Yang W. T. (2008). Insights into current limitations of density functional theory. Science 321, 792–794. 10.1126/science.1158722 18687952

[B10] Dan-Hardi M. Serre C. Frot T. Rozes L. Maurin G. Sanchez C.. (2009). A new photoactive crystalline highly porous titanium(IV) dicarboxylate. J. Am. Chem. Soc. 131, 10857–10859. 10.1021/ja903726m 19621926

[B11] Dolg M. Wedig U. Stoll H. Preuss H. (1987). Energy-adjusted ab initio pseudopotentials for the first row transition elements. J. Chem. Phys. 86, 866–872. 10.1063/1.452288

[B12] Dubbeldam D. Calero S. Ellis D. E. Snurr R. Q. (2015). Raspa: molecular simulation software for adsorption and diffusion in flexible nanoporous materials. Mol. Simul. 42, 81–101. 10.1080/08927022.2015.1010082

[B13] Ewing C. S. Hartmann M. J. Martin K. R. Musto A. M. Padinjarekutt S. J. Weiss E. M. (2015). Structural and electronic properties of Pt_13_ nanoclusters on amorphous silica supports. J. Phys. Chem. C 119, 2503–2512. 10.1021/jp5105104

[B14] Fampiou I. Ramasubramaniam A. (2012). Binding of Pt nanoclusters to point defects in graphene: adsorption, morphology, and electronic structure. J. Phys. Chem. C 116, 6543–6555. 10.1021/jp2110117

[B15] Fang X. Shang Q. Wang Y. Jiao L. Yao T. Li Y.. (2018). Single Pt atoms confined into a metal-organic framework for efficient photocatalysis. Adv. Mater. 30:1705112. 10.1002/adma.201705112 29315871

[B16] Finazzi E. Di Valentin C. Pacchioni G. Selloni A. (2008). Excess electron states in reduced bulk anatase TiO_2_: comparison of standard GGA, GGA+U, and hybrid DFT calculations. J. Chem. Phys. 129:154113. 10.1063/1.2996362 19045182

[B17] Frisch M. J. Trucks G. W. Schlegel H. B. Scuseria G. E. Robb M. A. Cheeseman J. R. (2013). Gaussian 09, Revision E.01. Wallingford, CT: Gaussian Inc.

[B18] Fu C. F. Wu X. J. Yang J. L. (2018). Material design for photocatalytic water splitting from a theoretical perspective. Adv. Mater. 30:1802106. 10.1002/adma.201802106 30328641

[B19] Fu Q. Colmenares Rausseo L. C. Martinez U. Dahl P. I. Garcia Lastra J. M. Vullum P. E.. (2015). Effect of Sb segregation on conductance and catalytic activity at Pt/Sb-doped SnO_2_ interface: a synergetic computational and experimental study. ACS Appl. Mater. Interfaces 7, 27782–27795. 10.1021/acsami.5b08966 26615834

[B20] Fu Y. H. Sun D. R. Chen Y. J. Huang R. K. Ding Z. X. Fu X. Z.. (2012). An amine-functionalized titanium metal-organic framework photocatalyst with visible-light-induced activity for CO_2_ reduction. Angew. Chem. Int. Ed. 51, 3364–3367. 10.1002/anie.201108357 22359408

[B21] Fujishima A. Honda K. (1972). Electrochemical photolysis of water at a semiconductor electrode. Nature 238, 37–38. 10.1038/238037a0 12635268

[B22] Furthmüller J. Kresse G. (1996). Efficient iterative schemes for ab initio total-energy calculations using a plane-wave basis set. Phys. Rev. B 54, 11169–11186. 10.1103/PhysRevB.54.11169 9984901

[B23] Gao W. W. Abtew T. A. Cai T. Y. Sun Y. Y. Zhang S. B. Zhang P. H. (2016). On the applicability of hybrid functionals for predicting fundamental properties of metals. Solid State Commun. 234, 10–13. 10.1016/j.ssc.2016.02.014

[B24] Han S. Y. Pan D. L. Chen H. Bu X. B. Gao Y. X. Gao H.. (2018). A methylthio-functionalized-mof photocatalyst with high performance for visible-light-driven H_2_ evolution. Angew. Chem. Int. Ed. 57, 9864–9869. 10.1002/anie.201806077 29898244

[B25] He J. Wang J. Chen Y. Zhang J. Duan D. Wang Y.. (2014). A dye-sensitized Pt@UiO-66(Zr) metal-organic framework for visible-light photocatalytic hydrogen production. Chem. Commun. 50, 7063–7066. 10.1039/C4CC01086H 24848342

[B26] He Y. Huang G. An C. Huang J. Zhang P. Chen X.. (2018). Reduction of *Escherichia coli* using ceramic disk filter decorated by nano-TiO_2_: a low-cost solution for household water purification. Sci. Total Environ. 616–617, 1628–1637. 10.1016/j.scitotenv.2017.10.149 29066198

[B27] Hendon C. H. Tiana D. Fontecave M. Sanchez C. D'Arras L. Sassoye C.. (2013). Engineering the optical response of the titanium-MIL-125 metal-organic framework through ligand functionalization. J. Am. Chem. Soc. 135, 10942–10945. 10.1021/ja405350u 23841821

[B28] Hendrickx K. Vanpoucke D. E. Leus K. Lejaeghere K. Van Yperen-De Deyne A. Van Speybroeck V.. (2015). Understanding intrinsic light absorption properties of UiO-66 frameworks: a combined theoretical and experimental study. Inorg. Chem. 54, 10701–10710. 10.1021/acs.inorgchem.5b01593 26540517

[B29] Hess B. (2000). Similarities between principal components of protein dynamics and random diffusion. Phys. Rev. E 62, 8438–8448. 10.1103/PhysRevE.62.8438 11138145

[B30] Ingler W. B. Jr. Baltrus J. P. Khan S. U. (2004). Photoresponse of p-type zinc-doped iron(III) oxide thin films. J. Am. Chem. Soc. 126, 10238–10239. 10.1021/ja048461y 15315424

[B31] Kadantsev E. S. Boyd P. G. Daff T. D. Woo T. K. (2013). Fast and accurate electrostatics in metal organic frameworks with a robust charge equilibration parameterization for high-throughput virtual screening of gas adsorption. J. Phys. Chem. Lett. 4, 3056–3061. 10.1021/jz401479k

[B32] Kasap H. Caputo C. A. Martindale B. C. M. Godin R. Lau V. W.-H. Lotsch B. V.. (2016). Solar-driven reduction of aqueous protons coupled to selective alcohol oxidation with a carbon nitride–molecular Ni catalyst system. J. Am. Chem. Soc. 138, 9183–9192. 10.1021/jacs.6b04325 27337491 PMC4965840

[B33] Kresse G. Joubert D. (1999). From ultrasoft pseudopotentials to the projector augmented-wave method. Phys. Rev. B 59, 1758–1775. 10.1103/PhysRevB.59.1758

[B34] Krukau A. V. Vydrov O. A. Izmaylov A. F. Scuseria G. E. (2006). Influence of the exchange screening parameter on the performance of screened hybrid functionals. J. Chem. Phys. 125:224106. 10.1063/1.2404663 17176133

[B35] Kumaravel V. Mathew S. Bartlett J. Pillai S. C. (2019). Photocatalytic hydrogen production using metal doped TiO_2_: A review of recent advances. Appl. Catal. B Environ. 244, 1021–1064. 10.1016/j.apcatb.2018.11.080

[B36] Kwon S. Fan M. H. Cooper A. T. Yang H. Q. (2008). Photocatalytic applications of micro- and nano-TiO_2_ in environmental engineering. Crit. Rev. Env. Sci. Tec. 38, 197–226. 10.1080/10643380701628933

[B37] Lian Z. C. Wang W. C. Li G. S. Tian F. H. Schanze K. S. Li H. X. (2017). Pt-enhanced mesoporous Ti3+/ TiO_2_ with rapid bulk to surface electron transfer for photocatalytic hydrogen evolution. ACS Appl. Mater. Interfaces 9, 16959–16966. 10.1021/acsami.6b11494 28001032

[B38] Lim D.-H. Wilcox J. (2011). DFT-based study on oxygen adsorption on defective graphene-supported Pt nanoparticles. J. Phys. Chem. C 115, 22742–22747. 10.1021/jp205244m

[B39] Liu H. Xu C. Y. Li D. D. Jiang H. L. (2018). Photocatalytic hydrogen production coupled with selective benzylamine oxidation over MOF composites. Angew. Chem. Int. Ed. 57, 5379–5383. 10.1002/anie.201800320 29508919

[B40] Liu H. H. Tian K. F. Ning J. Q. Zhong Y. J. Zhang Z. Y. Hu Y. (2019). One-step solvothermal formation of pt nanoparticles decorated Pt^2+^-doped α-Fe_2_O_3_ nanoplates with enhanced photocatalytic O_2_ evolution. ACS Catal. 9, 1211–1219. 10.1021/acscatal.8b03819

[B41] Liu J. J. Cheng B. Yu J. G. (2016). A new understanding of the photocatalytic mechanism of the direct Z-scheme g-C_3_N_4_/TiO_2_ heterostructure. Phys. Chem. Chem. Phys. 18, 31175–31183. 10.1039/C6CP06147H 27819105

[B42] Lu T. Chen F. W. (2012). Multiwfn: a multifunctional wavefunction analyzer. J. Comput. Chem. 33, 580–592. 10.1002/jcc.22885 22162017

[B43] Ma X. L. Liu S. G. Huang S. P. (2017). Hydrogen adsorption and dissociation on the Tm-doped (Tm=Ti, Nb) Mg_55_ nanoclusters: a DFT study. Int. J. Hydrogen Energy 42, 24797–24810. 10.1016/j.ijhydene.2017.08.086

[B44] Maisuradze G. G. Liwo A. Scheraga H. A. (2009). Principal component analysis for protein folding dynamics. J. Mol. Biol. 385, 312–329. 10.1016/j.jmb.2008.10.018 18952103 PMC2652707

[B45] Nasalevich M. A. Hendon C. H. Santaclara J. G. Svane K. van der Linden B. Veber S. L. (2016). Electronic origins of photocatalytic activity in *d*^0^ metal organic frameworks. Sci. Rep. 6:23676 10.1038/srep23676 27020767 PMC4810359

[B46] Neugebauer J. Scheffler M. (1992). Adsorbate-substrate and adsorbate-adsorbate interactions of Na and K adlayers on Al(111). Phys. Rev. B 46, 16067–16080. 10.1103/PhysRevB.46.16067 10003746

[B47] Ni B. L. Cai X. Lin J. Li Y. Huang S. P. Li Z. H. (2018). Tailoring the linear and second-order nonlinear optical responses of the titanium-MIL-125 metal–organic framework through ligand functionalization: a first principles study. J. Phys. Chem. C 123, 653–664. 10.1021/acs.jpcc.8b08008

[B48] Perdew J. P. Burke K. Ernzerhof M. (1996). Generalized gradient approximation made simple. Phys. Rev. Lett. 77, 3865–3868. 10.1103/PhysRevLett.77.3865 10062328

[B49] Ramos-Sanchez G. Balbuena P. B. (2013). Interactions of platinum clusters with a graphite substrate. Phys. Chem. Chem. Phys. 15, 11950–11959. 10.1039/c3cp51791h 23771184

[B50] Ran J. R. Guo W. W. Wang H. L. Zhu B. C. Yu J. G. Qiao S. Z. (2018). Metal-free 2D/2D phosphorene/g-C_3_N_4_ van der waals heterojunction for highly enhanced visible-light photocatalytic H_2_ production. Adv. Mater. 30:1800128. 10.1002/adma.201800128 29707838

[B51] Rappé A. K. Casewit C. J. Colwell K. S. III. Goddard W. A. Skiff W. M. (1992). UFF, a full periodic table force field for molecular mechanics and molecular dynamics simulations. J. Am. Chem. Soc. 114, 10024–10035. 10.1021/ja00051a040

[B52] Ren Z. B. Liu N. Chen B. H. Li J. W. Mei D. H. (2018). Theoretical investigation of the structural stabilities of ceria surfaces and supported metal nanocluster in vapor and aqueous phases. J. Phys. Chem. C 122, 4828–4840. 10.1021/acs.jpcc.7b10208

[B53] Runge E. Gross E. K. U. (1984). Density-functional theory for time-dependent systems. Phys. Rev. Lett. 52, 997–1000. 10.1103/PhysRevLett.52.997

[B54] Rusu P. C. Brocks G. (2006). Surface dipoles and work functions of alkylthiolates and fluorinated alkylthiolates on Au(111). J. Phys. Chem. B 110, 22628–22634. 10.1021/jp0642847 17092010

[B55] Sakurai M. Watanabe K. Sumiyama K. Suzuki K. (1999). Magic numbers in transition metal (Fe, Ti, Zr, Nb, and Ta) clusters observed by time-of-flight mass spectrometry. J. Chem. Phys. 111, 235–238. 10.1063/1.479268

[B56] Scanlon D. O. Dunnill C. W. Buckeridge J. Shevlin S. A. Logsdail A. J. Woodley S. M.. (2013). Band alignment of rutile and anatase TiO_2_. Nat. Mater. 12, 798–801. 10.1038/nmat3697 23832124

[B57] Shen L. Luo M. Huang L. Feng P. Wu L. (2015). A clean and general strategy to decorate a titanium metal-organic framework with noble-metal nanoparticles for versatile photocatalytic applications. Inorg. Chem. 54, 1191–1193. 10.1021/ic502609a 25594784

[B58] Simon T. Bouchonville N. Berr M. J. Vaneski A. Adrović A. Volbers D.. (2014). Redox shuttle mechanism enhances photocatalytic H_2_ generation on Ni-decorated CdS nanorods. Nat. Mater. 13, 1013–1018. 10.1038/nmat4049 25087066

[B59] Singh R. Dutta S. (2018). A review on H_2_ production through photocatalytic reactions using TiO_2_/TiO_2_-assisted catalysts. Fuel 220, 607–620. 10.1016/j.fuel.2018.02.068

[B60] Toyao T. Saito M. Horiuchi Y. Mochizuki K. Iwata M. Higashimura H. (2013). Efficient hydrogen production and photocatalytic reduction of nitrobenzene over a visible-light-responsive metal–organic framework photocatalyst. Catal. Sci. Technol. 3, 2092–2097. 10.1039/c3cy00211j

[B61] Tung R. T. (2000). Chemical bonding and fermi level pinning at metal-semiconductor interfaces. Phys. Rev. Lett. 84, 6078–6081. 10.1103/PhysRevLett.84.6078 10991128

[B62] Vilhelmsen L. B. Walton K. S. Sholl D. S. (2012). Structure and mobility of metal clusters in mofs: Au, pd, and aupd clusters in mof-74. J. Am. Chem. Soc. 134, 12807–12816. 10.1021/ja305004a 22734664

[B63] Wang D. Liu Z. P. Yang W. M. (2017). Proton-promoted electron transfer in photocatalysis: key step for photocatalytic hydrogen evolution on metal/titania composites. ACS Catal. 7, 2744–2752. 10.1021/acscatal.7b00225

[B64] Wang H. Zhang L. Chen Z. Hu J. Li S. Wang Z.. (2014). Semiconductor heterojunction photocatalysts: design, construction, and photocatalytic performances. Chem. Soc. Rev. 43, 5234–5244. 10.1039/C4CS00126E 24841176

[B65] Wang Y. Xiang B. Yang H. Q. Hu C. W. (2017). Density functional theory study on the nucleation and growth of Pt_n_ clusters on γ-Al_2_O_3_(001) surface. ACS Omega 2, 3250–3259. 10.1021/acsomega.7b00342 31457650 PMC6641230

[B66] Wang Z. Li C. Domen K. (2019). Recent developments in heterogeneous photocatalysts for solar-driven overall water splitting. Chem. Soc. Rev. 48, 2109–2125. 10.1039/C8CS00542G 30328438

[B67] Weigend F. Ahlrichs R. (2005). Balanced basis sets of split valence, triple zeta valence and quadruple zeta valence quality for H to Rn: design and assessment of accuracy. Phys. Chem. Chem. Phys. 7, 3297–3305. 10.1039/b508541a 16240044

[B68] Wold S. Esbensen K. Geladi P. (1987). Principal component analysis. Chemom. Intell. Lab. Syst. 2, 37–52. 10.1016/0169-7439(87)80084-9

[B69] Wu X. P. Gagliardi L. Truhlar D. G. (2018). Cerium metal-organic framework for photocatalysis. J. Am. Chem. Soc. 140, 7904–7912. 10.1021/jacs.8b03613 29807431

[B70] Wu Z. L. Wang C. H. Zhao B. Dong J. Lu F. Wang W. H.. (2016). A semi-conductive copper-organic framework with two types of photocatalytic activity. Angew.Chem. Int. Ed. 55, 4938–4942. 10.1002/anie.201508325 27079818

[B71] Xiao J. D. Jiang H. L. (2019). Metal-organic frameworks for photocatalysis and photothermal catalysis. Acc. Chem. Res. 52, 356–366. 10.1021/acs.accounts.8b00521 30571078

[B72] Xiao J. D. Shang Q. C. Xiong Y. J. Zhang Q. Luo Y. Yu S. H.. (2016). Boosting photocatalytic hydrogen production of ametal–organic framework decorated with platinum nanoparticles:the platinum location matters. Angew. Chem. Int. Ed. 55, 9389–9393. 10.1002/anie.201603990 27321732

[B73] Xing J. Jiang H. B. Chen J. F. Li Y. H. Wu L. Yang S. (2013). Active sites on hydrogen evolution photocatalyst. J. Mater. Chem. A 1, 15258–15264. 10.1039/c3ta13167j

[B74] Yang J. H. Wang D. E. Han H. X. Li C. (2013). Roles of cocatalysts in photocatalysis and photoelectrocatalysis. Acc. Chem. Res. 46, 1900–1909. 10.1021/ar300227e 23530781

[B75] Yang Z. X. Geng Z. X. Zhang Y. X. Wang J. L. Ma S. H. (2011). Improved oxygen reduction activity on the ih Cu@Pt core–shell nanoparticles. Chem. Phys. Lett. 513, 118–123. 10.1016/j.cplett.2011.07.088

[B76] Zhang J. Dolg M. (2015). ABCluster: the artificial bee colony algorithm for cluster global optimization. Phys. Chem. Chem. Phys. 17, 24173–24181. 10.1039/C5CP04060D 26327507

[B77] Zhang S. T. Li C. M. Yan H. Wei M. Evans D. G. Duan X. (2014). Density functional theory study on the metal–support interaction between Ru cluster and anatase TiO_2_(101) surface. J. Phys. Chem. C 118, 3514–3522. 10.1021/jp409627p

[B78] Zheng H. S. Mukherjee S. Gangopadhyay K. Gangopadhyay S. (2015). Ultrafine Pt nanoparticle induced doping/strain of single layer graphene: experimental corroboration between conduction and raman characteristics. J. Mater. Sci. Mater. Electron. 26, 4746–4753. 10.1007/s10854-015-3043-y

[B79] Zhu S. Chen X. F. Li Z. C. Ye X. Y. Liu Y. Chen Y. (2020). Cooperation between inside and outside of TiO_2_: lattice Cu^+^ accelerates carrier migration to the surface of metal copper for photocatalytic CO_2_ reduction. Appl. Catal. B Environ. 264:118515 10.1016/j.apcatb.2019.118515

