# Supplementary material for: A Ti-MOF Decorated With a Pt Nanoparticle Cocatalyst for Efficient Photocatalytic H2 Evolution: A Theoretical Study
Source: Front Chem. 2020 Aug 7;8:660. doi: 10.3389/fchem.2020.00660 (PMC7427410; doi:10.3389/fchem.2020.00660)
Supplement: Supplementary file 1 [file Table_1.DOCX]

***Supplementary Material***

**CONTENTS**

**Figure S1**. The structure of H_2_BDC, H_2_BDC(SCH_3_)_2_, MIL-125, 20%-MIL-125-(SCH_3_)_2_.

**Figure S2**. The structure of Ih Pt_13_ cluster.

**Figure S3**. The model that some atoms at the bottom of slab were fixed during the surface optimization.

**Figure S4**. Convergence test calculations in the searching the most probable structure of Pt13 adsorbed on the 20%-MIL-125-(SCH3)2 (001) surface.

**Figure S5**. Band alignment diagram of CBM and VBM for 20%-MIL-125-(SCH3)2. The blue and pink dashed lines represent energy levels corresponding to redox potentials for water splitting (pH = 7; T= 298.15 K).

**Figure S6.** Top view of the initial structure of Pt_13_/20%-MIL-125-(SCH_3_)_2_. The Pt_13_ cluster is located at the center of the surface.

**Figure S7.** The DOS of Pt_13_ cluster and Pt_13_/20%-MIL-125-(SCH_3_)_2_ calculated using the HSE06 functional.

**Figure S8.** The band tail states formed in the Pt_13_/20%-MIL-125-(SCH_3_)_2_.

**Figure S9.** Proton adsorption on different sites of Pt_13_ cluster.

**Figure S10**. The electron transfer between Pt_13_ cluster and the 20%-MIL-125-(SCH_3_)_2_ (001) surface.

**Table S1.** The Bader charge and net charge of absorbed Pt_13_ cluster.

**Scheme S1.** The whole process for the screening.


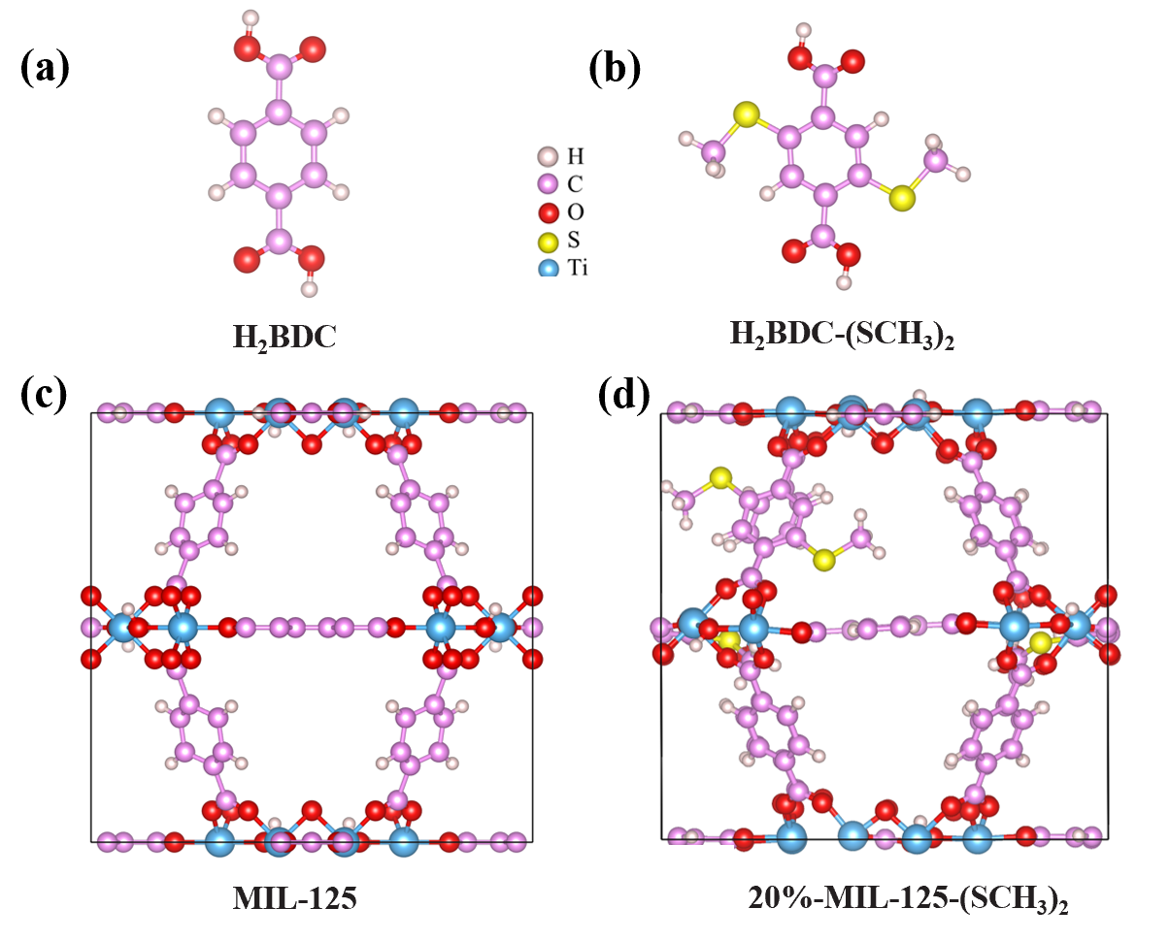


**Figure S1**. The structure of (a) H_2_BDC, (b) H_2_BDC(SCH_3_)_2_, (c) MIL-125, (d) 20%-MIL-125-(SCH_3_)_2_.


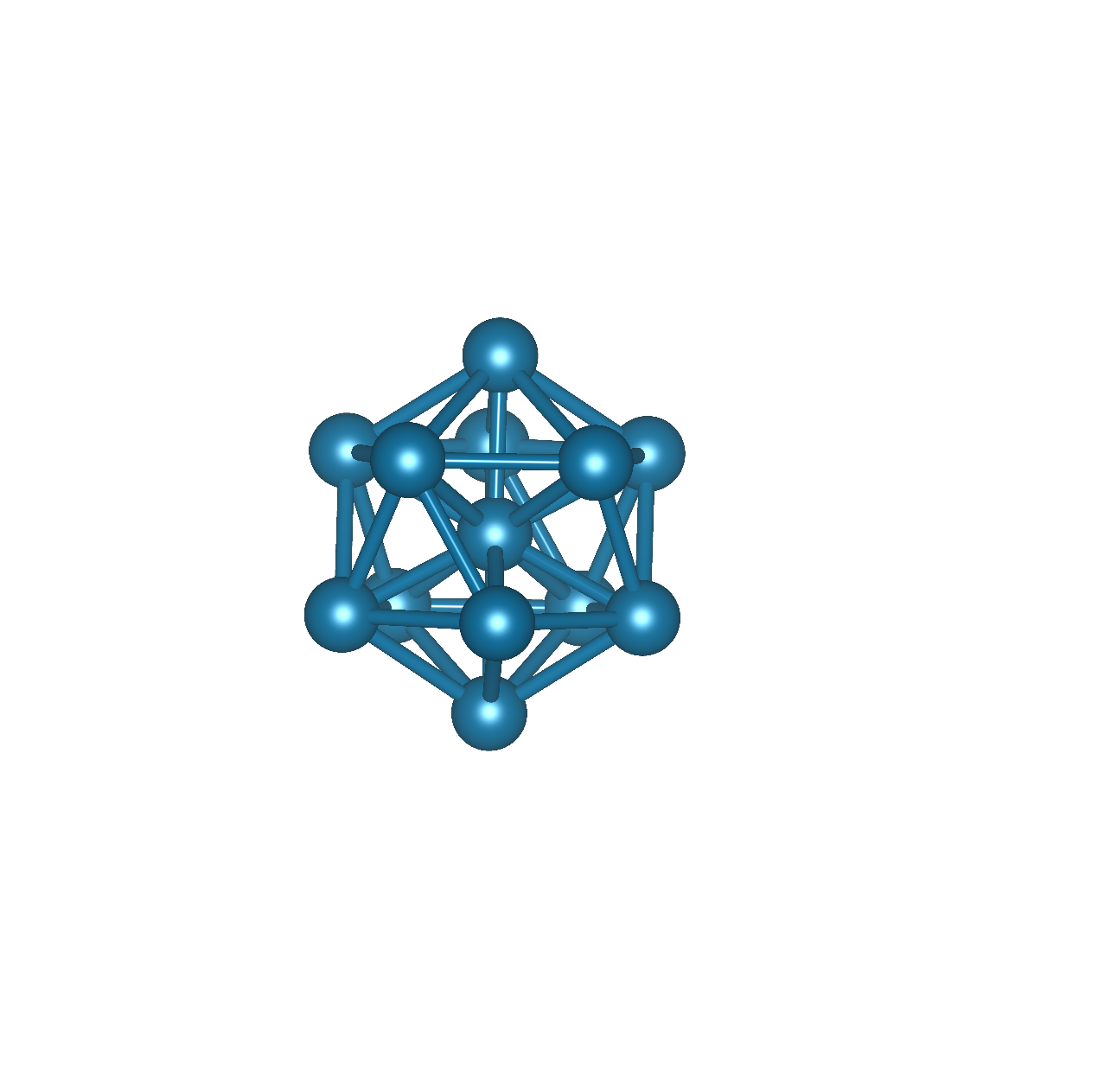


**Figure S2**. The structure of Ih Pt_13_ cluster.


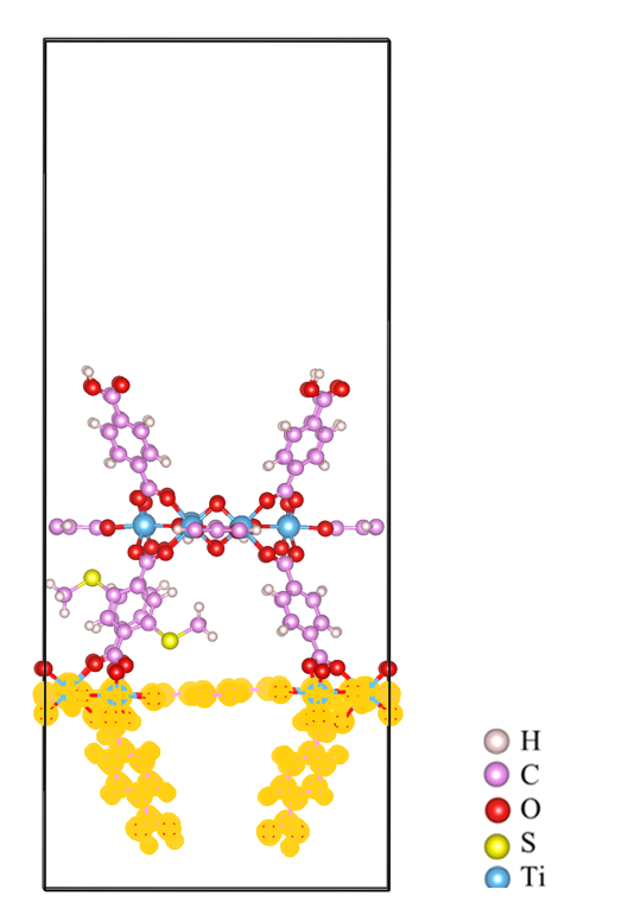


**Figure S3**. Atoms colored by yellow are fixed during the geometry optimization.


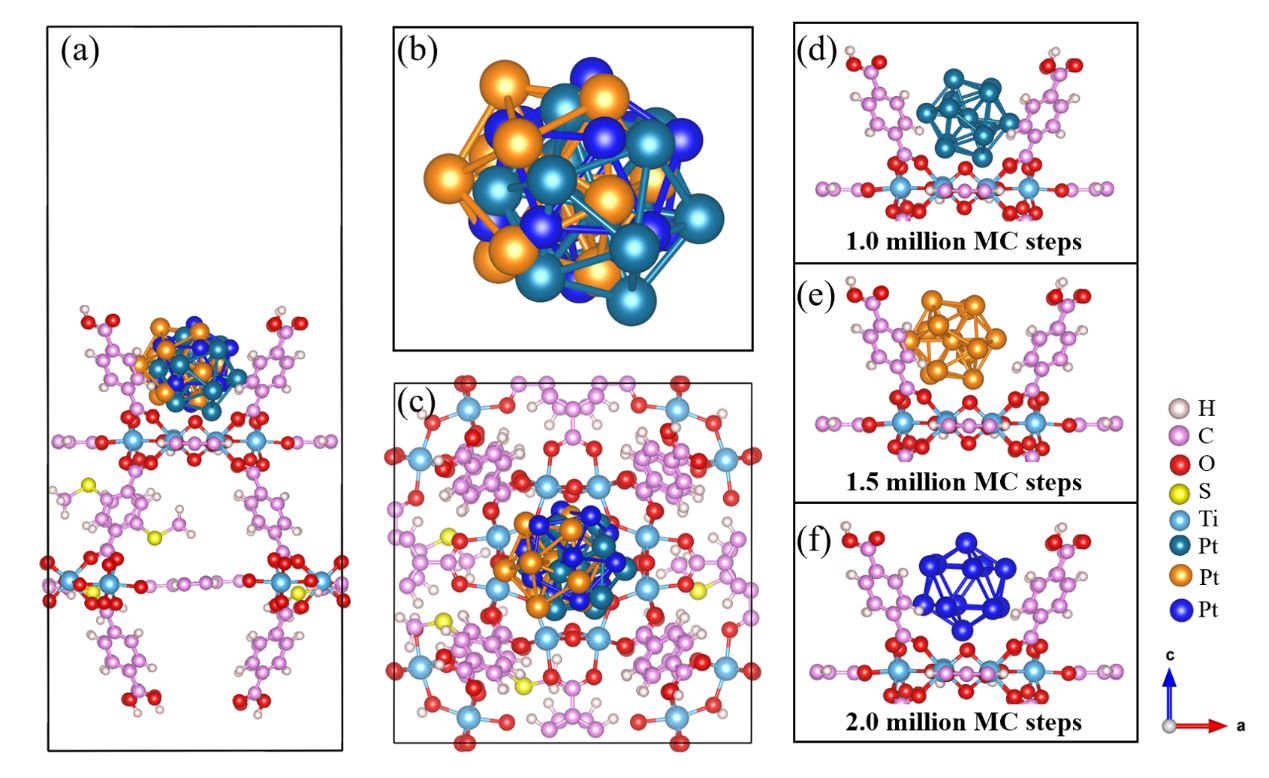


**Figure S4**. Convergence test calculations in the searching the most probable structure of Pt_13_ adsorbed on the 20%-MIL-125-(SCH_3_)_2_ (001) surface. (a)The obtained Pt_13_/20%-MIL-125-(SCH_3_)_2_ composite structures with different MC simulation steps; (b) conformation for Pt13 in different simulation steps; (c) top view for three obtained composite structures; (d-f) Zoom-in view for all three structures.


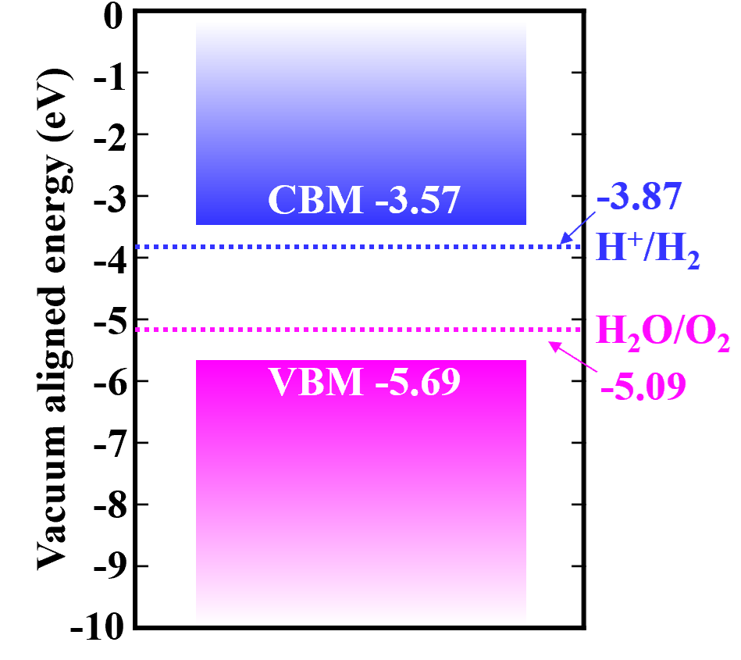


**Figure S5**. Band alignment diagram of CBM and VBM for 20%-MIL-125-(SCH3)2. The blue and pink dashed lines represent energy levels corresponding to redox potentials for water splitting (pH = 7; T= 298.15 K).


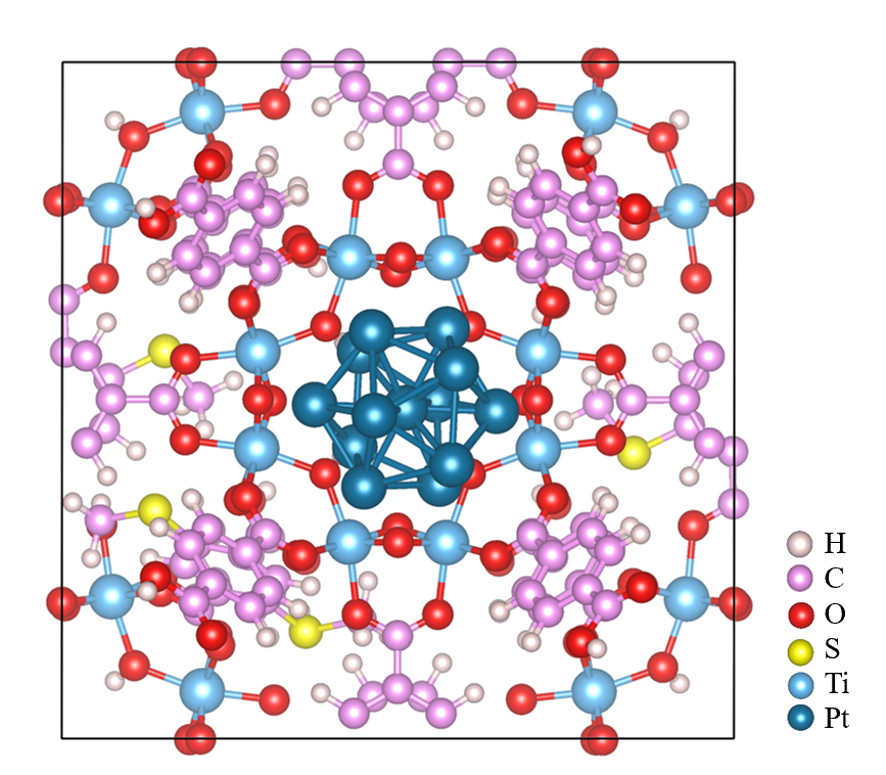


**Figure S6.** Top view of the initial structure of Pt_13_/20%-MIL-125-(SCH_3_)_2_. The Pt_13_ cluster is located at the center of the surface.


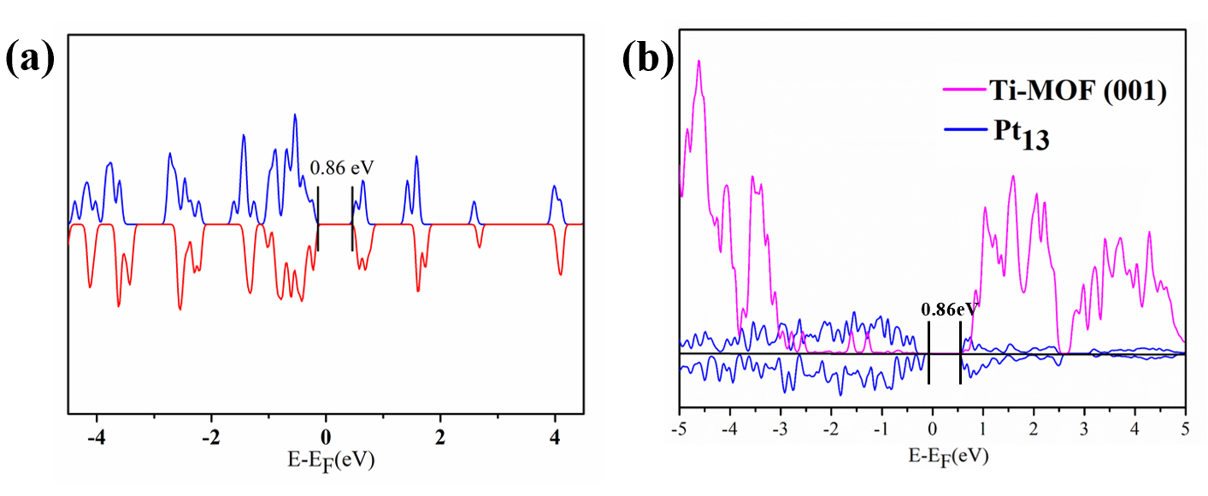


**Figure S7.** The DOS of Pt_13_ cluster and Pt_13_/20%-MIL-125-(SCH_3_)_2_ calculated using the HSE06 functional.


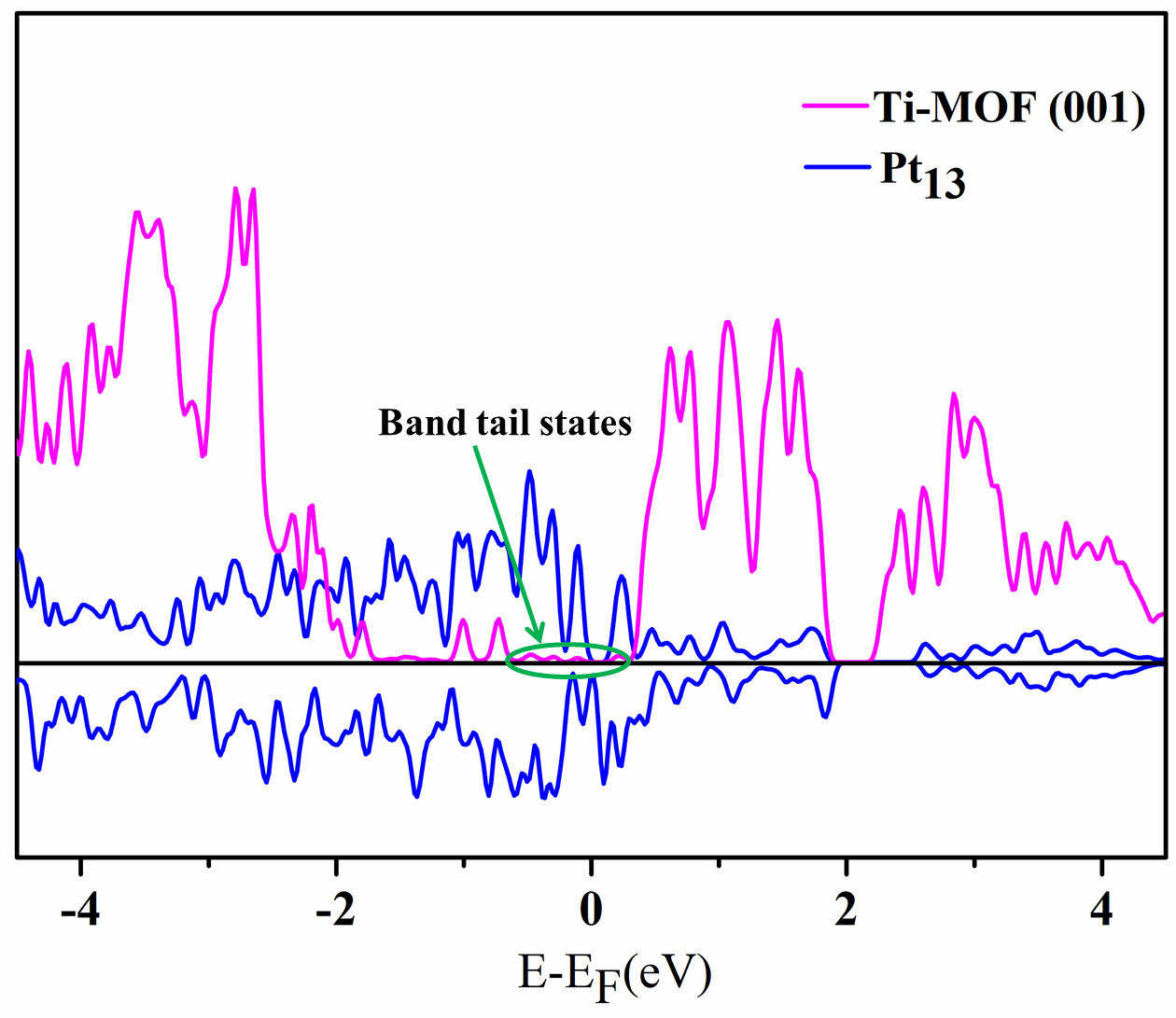


**Figure S8.** The band tail states formed in the Pt_13_/20%-MIL-125-(SCH_3_)_2_ composite system.


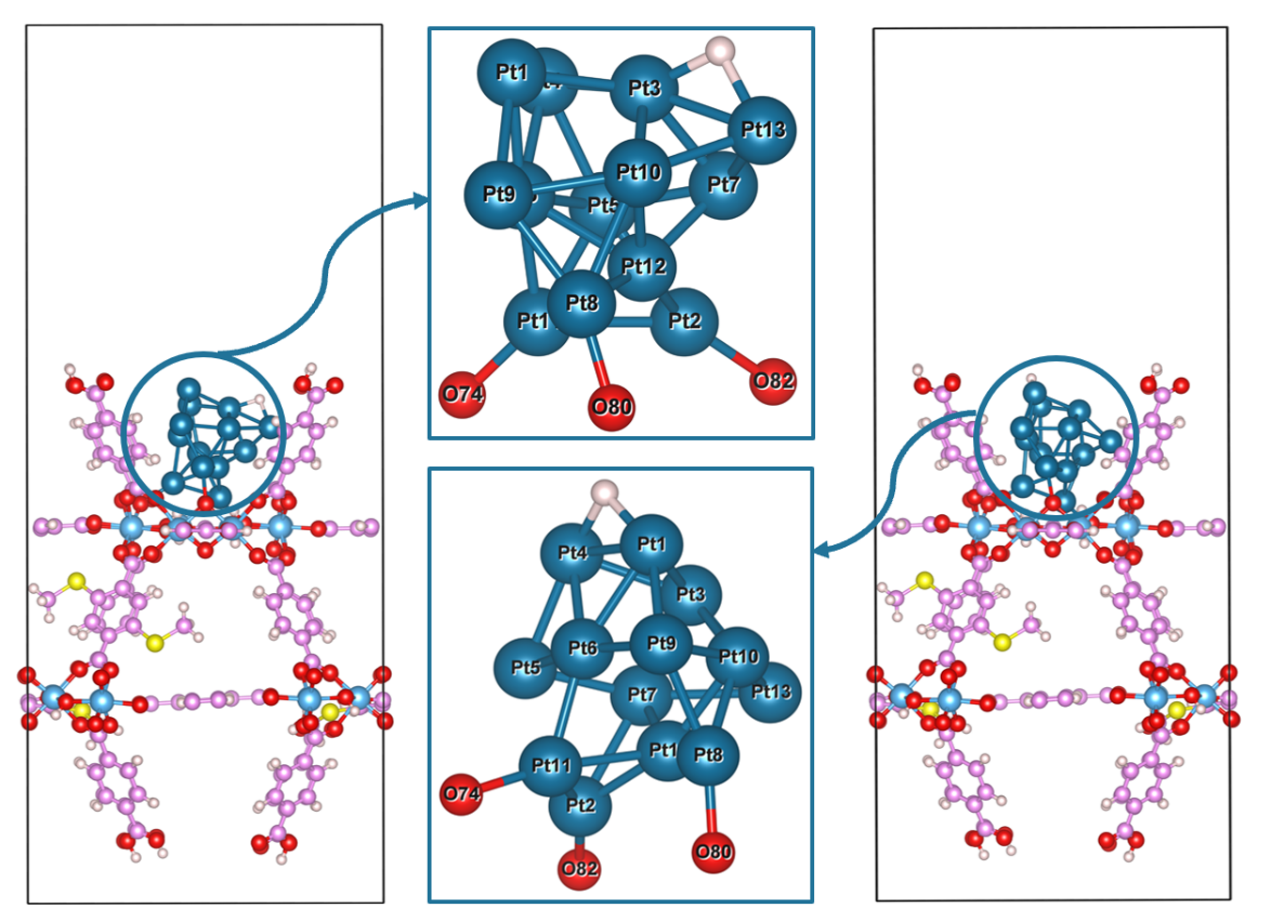


**Figure S9.** Proton adsorption on different sites of the Pt_13_ cluster.


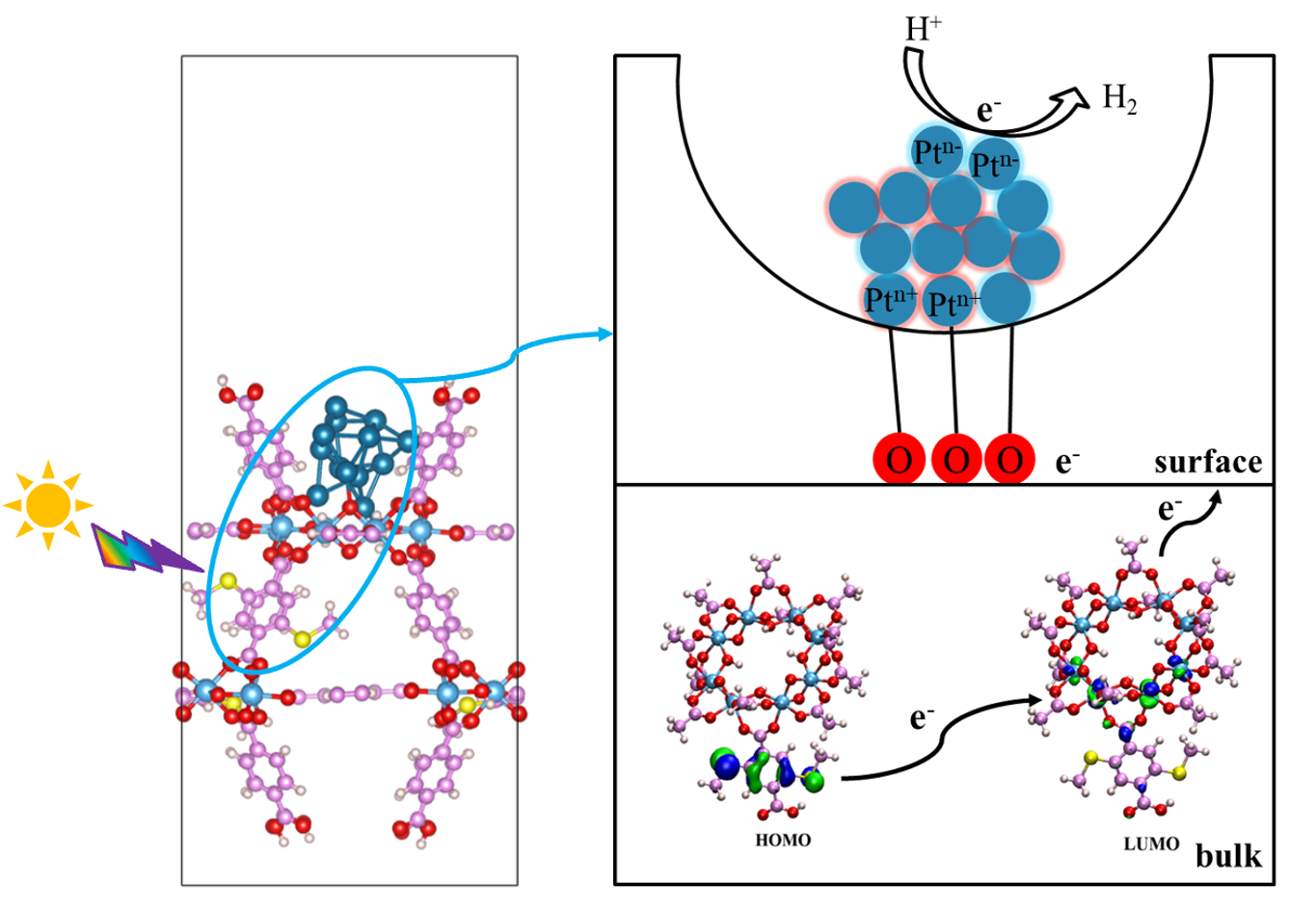


**Figure S10**. The electron transfer between Pt_13_ cluster and the 20%-MIL-125-(SCH_3_)_2_ (001) surface.

**Table S1.** The Bader charge and net charge of the absorbed Pt_13_ cluster. The symbol of the minus sign means getting electron.

| Pt atom number | Bader charge | Net charge |
| --- | --- | --- |
| 1 | 10.05 | -0.05 |
| 2 | 10.06 | -0.06 |
| 3 | 9.96 | 0.04 |
| 4 | 10.10 | -0.09 |
| 5 | 9.97 | 0.04 |
| 6 | 9.89 | 0.11 |
| 7 | 9.93 | 0.07 |
| 8 | 9.81 | 0.19 |
| 9 | 10.10 | -0.10 |
| 10 | 9.94 | 0.06 |
| 11 | 9.96 | 0.05 |
| 12 | 9.93 | 0.07 |
| 13 | 10.12 | -0.13 |
| Total charge | **129.80** | **0.20** |

**
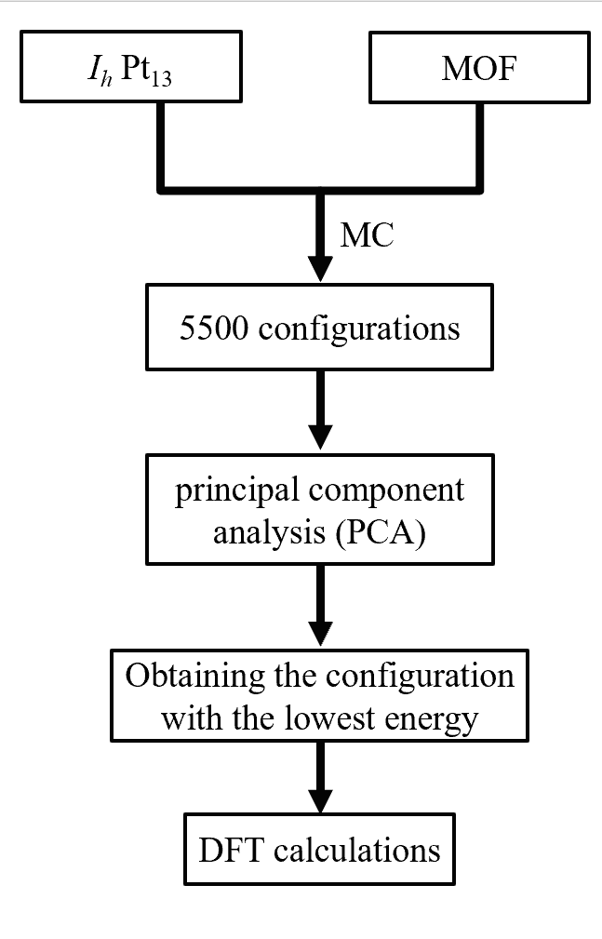
**

**Scheme S1.** The whole process for the screening.
